# Endocrine Disrupting Chemicals and Reproductive Health in Boys and Men

**DOI:** 10.3389/fendo.2021.706532

**Published:** 2021-10-07

**Authors:** Wiwat Rodprasert, Jorma Toppari, Helena E. Virtanen

**Affiliations:** ^1^ Research Centre for Integrative Physiology and Pharmacology, Institute of Biomedicine, University of Turku, Turku, Finland; ^2^ Centre for Population Health Research, University of Turku and Turku University Hospital, Turku, Finland; ^3^ Department of Pediatrics, Turku University Hospital, Turku, Finland

**Keywords:** anogenital distance, cryptorchidism, hypospadias, endocrine disrupters, endocrine disrupting chemicals, reproductive hormones, semen quality, testicular cancer

## Abstract

Male reproductive health has declined as indicated by increasing rates of cryptorchidism, i.e., undescended testis, poor semen quality, low serum testosterone level, and testicular cancer. Exposure to endocrine disrupting chemicals (EDCs) has been proposed to have a role in this finding. In utero exposure to antiandrogenic EDCs, particularly at a sensitive period of fetal testicular development, the so-called ‘masculinization programming window (MPW)’, can disturb testicular development and function. Low androgen effect during the MPW can cause both short- and long-term reproductive disorders. A concurrent exposure to EDCs may also affect testicular function or damage testicular cells. Evidence from animal studies supports the role of endocrine disrupting chemicals in development of male reproductive disorders. However, evidence from epidemiological studies is relatively mixed. In this article, we review the current literature that evaluated relationship between prenatal EDC exposures and anogenital distance, cryptorchidism, and congenital penile abnormality called hypospadias. We review also studies on the association between early life and postnatal EDC exposure and semen quality, hypothalamic-pituitary-gonadal axis hormone levels and testicular cancer.

## 1 Introduction

Reports on deteriorating male reproductive health have been published in many countries. Serum testosterone levels and semen quality have been declining (1–3). In addition, the rates of congenital cryptorchidism, i.e. undescended testis, and testicular germ cell tumors have been increasing (4, 5). Exposure to endocrine disrupting chemicals (EDCs) has been proposed to be one of the causes of these adverse trends. This is because these chemicals are ubiquitous, we are exposed to them *via* food, skin and inhaled air. Environmental EDCs include for instance pesticides, chemicals used in plastic products [like phthalates and bisphenol A (BPA)], in personal care products (like triclosan and parabens), in hydraulic and electronic devices [like polychlorinated biphenyls (PCBs)], chemicals used in clothes (like perfluorinated compounds), flame retardants, solvents, chemicals produced unintentionally as side products in chemical processes (dioxins) and many others (6).

**Figure 1:**
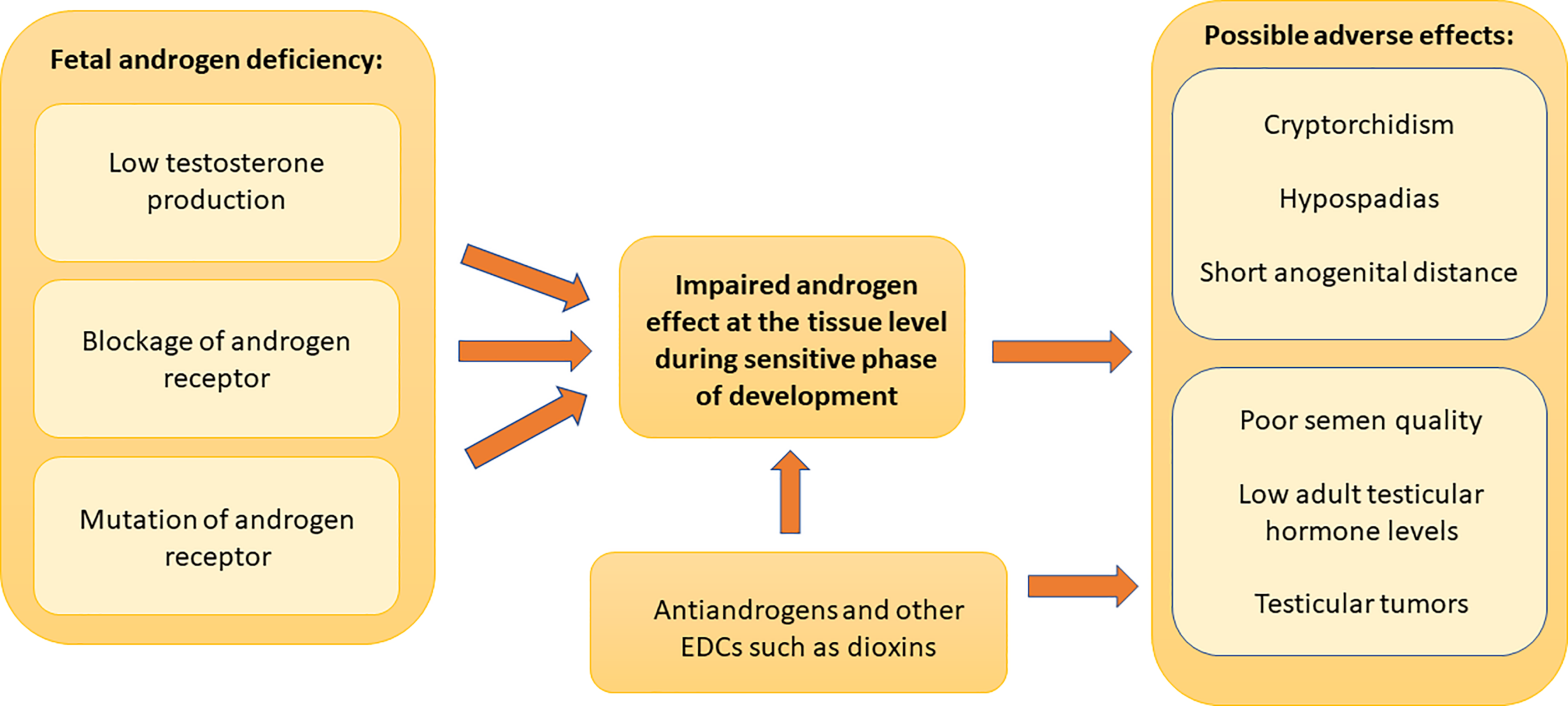
Role of androgen effects in male reproductive disorders. Adequate androgen action during a sensitive period of development in male fetus is important for normal male reproductive organ development and function after birth. The lack of androgen action due to decreased testosterone synthesis, androgen receptor blockade or androgen receptor mutations can cause early or late postnatal male reproductive disorders. Early postnatal manifestations include cryptorchidism, hypospadias and decreased anogenital distance. Late postnatal manifestations consist of reduced semen quality, reduced adult reproductive hormone levels and testicular germ cell tumors. Fetal exposure to chemicals that have antiandrogenic effects can disturb male reproductive system development and can cause these manifestations. These chemicals can also cause postnatal antiandrogenic effects as shown by the direct arrow to the adult manifestations. There are also other possible mechanisms of action, and other endocrine disrupting compounds may also affect reproductive organs. Dioxin is a well-known example of such a chemical.

Many experimental and epidemiological studies have supported links between EDC exposures and male reproductive health problems (7). Since development of male reproductive system requires androgens, substances that have antiandrogenic effects can disturb this process and possibly cause male reproductive disorders **(** **Figure 1** **)**. Anti-androgenic chemicals with different mechanisms of actions (inhibition of androgen biosynthesis or receptor antagonism) show accumulative effects (8). This causes the risk that even low concentrations in mixtures can be harmful. Furthermore, non-monotonic dose-response to EDCs has been described (9, 10).

It has been proposed that the disruption of fetal testicular development due to, for example, maternal exposure to EDCs, can result in disorders manifested at birth, i.e., congenital cryptorchidism, congenital penile abnormality called hypospadias and reduced anogenital distance (AGD), as well as disorders presented later in life, including poor semen quality, testicular germ cell tumors, and altered reproductive hormone levels. This is the concept of testicular dysgenesis syndrome, TDS (11, 12). In addition, some studies have shown associations between postnatal EDC exposures and male reproductive disorders.

We will review the human epidemiological studies that investigated the association between pre- and postnatal EDC exposure (based on environmental chemical concentration measurements in different matrices) and above mentioned male reproductive health indicators (anogenital distance, cryptorchidism, hypospadias, semen quality, reproductive hormone levels in adults and testicular cancer) and were published in English by August 2020 and found in Pubmed. Heavy metals and pharmaceuticals are not included in this review, because medicines have been recently reviewed elsewhere (13) and because the effects of heavy metals are mostly toxic rather than endocrine modulating (14, 15). However, we include organotins, because their action is clearly hormonal.

### 1.1 Short Introduction to EDCs

EDCs can disturb hormonal systems and may cause male reproductive disorders by a variety of mechanisms. Studies have shown that EDCs can have estrogenic, anti-estrogenic, androgenic or antiandrogenic effects (16). PCBs, polybrominated diphenyl ethers (PBDEs), phthalates, and bisphenol A can act on estrogen receptor and exert estrogenic effects (7, 16) In contrast, benzophenone-3 and -4 and some PCBs showed antiestrogenic effects. Some ultraviolet (UV) filters, BPA, p,p′-dichlorodiphenyldichloroethylene (p,p’-DDE), PBDEs and phthalates have antiandrogenic activity (16–20). PCB-138, -153, -180, have pleiotropic effects on androgen and estrogen receptors (19, 20). Organochlorine compounds, including polychlorinated dibenzo-p-dioxins, dichlorodiphenyltrichloroethane (DDT), hexachlorobenzene (HCB) and PCBs, can bind to estrogen receptors and exert estrogenic effects or have antiandrogenic effects (16, 21–23). Only few EDCs have been reported to have androgenic activity, for example, benzophenone 2 (16). Dioxins can also bind to aryl hydrocarbon receptor (AhR), which functions in association with estrogen or androgen receptor (7, 24). Lastly, some EDCs can directly disturb spermatogenesis and cause poor semen quality.

#### 1.1.1 Persistent EDCs

EDCs include persistent and non-persistent chemicals. Persistent organic pollutants include chemicals that can accumulate and are persistent in the body or environment. PCBs and DDT, are examples of lipophilic chemicals that can accumulate in adipose tissue, are slowly excreted, and therefore they can persist in the body for a long time (25). Because of the long half-life, the adult levels of these chemicals can be used to study an association with prenatal exposure, although the timing of exposure is unclear.

##### 1.1.1.1 Pesticides

Dichlordiphenyldichloroethylene (p,p’-DDE) is the most persistent congener of DDT. The effects of DDE and DDT may persist even though they were banned in 1970s-1980s (26, 27). DDT and p,p’-DDE can accumulate in body fat for many years (half-life of approximately 6 years for DDT and 10 years for p,p’-DDE) (6, 27, 28). Persistent chemicals include also other organochlorine pesticides, for example lindane, chlordane and heptachlor (25).

##### 1.1.1.2 PCBs and Dioxins

PCBs were widely used in industrial and consumer products. Even though their use was banned in the 1970s, they still persist in the environment and people continue to be exposed (29). They accumulate in body fat and have a half-life of 1 to 10 years. Humans are exposed to PCBs through ingestion of contaminated food, inhalation or skin contact (29). As mentioned above, dioxins are not produced intentionally, but they are formed as side products and humans are exposed to these persistent chemicals mainly *via* food of animal origin (30).

##### 1.1.1.3 Flame Retardants

PBDEs are used as flame retardants and are found in house dust. The major routes of exposure are dust inhalation or ingestion (31, 32). They can exert anti-androgenic and estrogenic activity, which potentially leads to male reproductive disorders (16, 33). Also polybrominated biphenyls (PBBs) have been used as flame retardants (6).

##### 1.1.1.4 Perfluorinated Compounds

Perfluorinated compounds (PFCs) are used in industry and consumer products, including surfactants, paints, lubricants and impregnation of clothes, textiles, footwear, furniture and carpets (34). Perfluorooctane sulfonate (PFOS) is the most abundant perfluoroalkyl substances (PFAS) in humans and in environment, followed by perfluorooctanoic acid (PFOA) (35, 36). PFOA was used in the production of polytetrafluoroethylene, which is used in non-stick coating cookware (37). Human exposure occurs *via* inhalation, ingestion and skin contact (38).

##### 1.1.1.5 Organotins

Organotins have been used widely in industry, e.g., in anti-fouling paints of boats and ships and they have been observed to have endocrine-disrupting properties and adverse effect on male reproductive health (6, 39). Humans are exposed to them *via* contaminated seafood.

#### 1.1.2 Non-Persistent EDCs

Non-persistent endocrine disrupting chemicals include, for example, BPA, parabens, triclosan, phthalates, synthetic pyrethroids and organophosphate pesticides (40).

##### 1.1.2.1 BPA and Other Phenols

Bisphenol A is used in the lining of water supply pipes, aluminum cans, reusable plastic food containers, dental sealants, thermal receipts, medical equipment, and building supplies (41). Humans can be exposed to BPA *via* ingestion, inhalation or skin contact (42). It can act as a weak agonist of the estrogen receptor by binding to estrogen receptors (ER) ERα and Erβ (43, 44). It can also act as an androgen receptor antagonist (45, 46). It can cause reduced serum follicle-stimulating hormone (FSH), luteinizing hormone (LH) and testosterone levels (47). It can interfere 17α-hydroxylase/17,20 lyase and aromatase, which are important steroidogenic enzymes of Leydig cells (46). In addition, it can cross the placenta from mothers to the fetus, but its concentration in fetal circulation is much lower than in mother and thus, the placenta appears to reduce BPA exposure of the fetus (48). BPA is metabolized in the liver and excreted in urine with plasma half-life of six hours (46). Therefore, the standard method of BPA measurement is analysis of urinary levels (42, 49). Bisphenol S was used as a potentially safer substitute for BPA. However, a limited number of studies showed that it also has estrogenic, androgenic, and anti-androgenic effects (50), and therefore it might have adverse reproductive effects in humans. Triclosan is an antimicrobial agent used for instance in personal care products and it is also a phenol (6).

##### 1.1.2.2 Phthalates

Phthalates are ubiquitous chemicals, which are widely used as plasticizers, a component of polyvinyl chloride (PVC), excipients in some medications, personal care products, solvents or adhesives (51). Humans are exposed to phthalates *via* ingestion, which is the main route of exposure, inhalation, intravenous administration and through direct skin contact (51). After entering the human body, phthalates are rapidly metabolized into monoesters, which are excreted into urine with a half-life of 12 hours (52, 53). Therefore, phthalate measurement from urine results in a higher level than from other biological samples, and urine is the most frequently used sample in epidemiological studies (54).

##### 1.1.2.3 Parabens

Parabens belong to a group of esters of p-hydroxybenzoic acid. They have antibacterial and antifungal properties, therefore they are used as preservatives in personal care products, cosmetics, foodstuffs and some pharmaceuticals (55–57). They show weak estrogenic effect *in vitro* (57). Parabens belong to non-halogenated phenols (6).

##### 1.1.2.4 Non-Persistent Pesticides

Non-persistent pesticides include for instance organophosphates, pyrethroids, and carbamates. Some of these chemicals have been shown to have endocrine disrupting effects and may cause male reproductive disorders (58, 59).

##### 1.1.2.5 Solvents

Solvents are widely present in occupational and consumer products, such as cleaning products and cosmetics. These chemicals include for instance glycol ethers, some of which have been shown to affect testicular function and expression of estrogen and androgen receptors in the testis (60, 61).

## 2 Reproductive outcomes

### 2.1 Anogenital Distance

Anogenital distance has been measured either as anoscrotal distance, i.e., the distance between anus and perineoscrotal junction, or as an anopenile distance, i.e., the distance between anus and cephalad insertion of the penis. Sometimes also the distance from the centre of the anus to the posterior base of the penis was recorded (62). Anogenital distance is considered to be a life-long marker of androgen exposure in the prenatal male programming window (MPW), at least in rats (63, 64). In humans, MPW is presumed to be in gestational weeks (GW) 8–14 (63). Prenatal exposure to antiandrogenic EDCs has been associated with short AGD in male rats [reviewed in (65)]. Several human studies have evaluated associations between prenatal EDC exposure and anogenital distance in infant and young boys (**Table 1**).

**Table 1:** Studies on the association between exposure to different classes of environmental EDCs (based on matrix measurements) and anogenital distance in young boys.

Reference	matrix	Chemicals/congeners analysed	n of subjects	Country	Association between chemical levels and AGD
**Dioxins**					
Vafeiadi (66)	Maternal plasma collected at delivery	Plasma dioxin-like activity	119 newborn boys, 239 young boys (median age 1.6 years)	Greece and Spain	Anopenile distance in newborns: Negative association with maternal plasma dioxin-like activity.
**Flame retardants**					
García-Villarino (67)	Cord blood	6 PBDEs	116 4-y old boys	Spain	PBDE-153 levels were associated positively with anoscrotal distance/weight
Luan (68)	Cord plasma	9 PBDEs	190 boys [measured at birth (n=182), at 6 mo (n=148), at 12 mo (n=149), or at 48 mo (n=158)]	China	Anoscrotal distance: Significant negative associations in the highest quartile of BDE-47 and sum of 4 PBDEs at 12 or 48 mo. Mid-range levels of BDE-28 were associated with shorter anopenile distance at 48 months of age.
García-Villarino et al. (69)	Maternal serum at first trimester of pregnancy	PBDE-28, -99, -153	27 18-mo-old boys	Spain	Anoscrotal distance/weight was negatively associated with PBDE-99 and PBDE-153 levels
García-Villarino (67)	Maternal serum at first trimester	6 PBDEs	74 4-y old boys	Spain	Levels of PBDE-209 were negatively associated with anoscrotal distance/weight
**Parabens**					
Fisher (70)	Maternal serum during pregnancy	6 parabens	237	UK	Detection of n-Propyl paraben was associated with shorter anoscrotal distance from birth to 24 mo of age
**PCBs**					
García-Villarino (67)	Cord blood	6 PCBs	116 4-y old boys	Spain	PCB-153 and -180 levels were negatively associated with anoscrotal distance/weight
García-Villarino (69)	Maternal serum at first trimester of pregnancy	PCB-28, -52	27 18-mo-old boys	Spain	NS
García-Villarino et al. (67)	Maternal serum at first trimester	6 PCBs	74 4-y old boys	Spain	PCB-138 (second tertile), -153 (second tertile), -180 levels were negatively associated with anoscrotal distance/weight
Loreto-Gómez et al. (62)	Maternal serum during third trimester of pregnancy	7 PCBs	74 boys, followed at 0, 1, 3, 6 and 12 mo	Mexico	Significant negative association between anopenile distance/height and PCB 28, 74, and 170 levels (individually and combined).
**Perfluorinated compounds**					
Arbuckle (71)	Maternal plasma during first trimester	PFOA, PFOS and PFHxS	205 newborn boys	Canada	PFOA levels showed positive association with anoscrotal distance, but no dose-response effect
Lind (72)	Maternal serum during first trimester	PFOS, PFOA, PFHxS, PFNA, and PFDA	316 boys examined 3 months after expected date of delivery	Denmark	No consistent association between PFASs levels and anopenile or anoscrotal distance
Tian (73)	Maternal plasma during pregnancy	Eleven PFASs	500 boys examined at least once at birth (n=439), at 6 (n=322) or at 12 months (n=301)	China	PFOS, PFDA, PFUdA and PFTrDA levels were negatively associated with anoscrotal or anopenile distance at 0 or at 6 months.
**Pesticides**					
García-Villarino (67)	cord blood	beta-HCH, gamma-HCH (lindane), HCB, 4,4′-DDT, 4,4′-DDE, 4,4′-DDD,	116 4-y old boys	Spain	NS
Bornman (74)	Maternal serum at delivery or after it	DDT, DDE	343 at newborn, 344 at 1 year (follow-up)	South Africa	NS
García-Villarino (69)	Maternal serum at first trimester of pregnancy	2,4-DDD, 4,4-DDD, HCB	27 18-mo-old boys	Spain	NS
García-Villarino et al. (67)	Maternal serum at first trimester	beta-HCH, gamma-HCH (lindane), HCB, 4,4′-DDT, 4,4′-DDE, 4,4′-DDD,	74 4-y old boys	Spain	NS
Longnecker (75)	Maternal serum postpartum	DDT, DDE	781 newly delivered infants	Mexico	NS
Loreto-Gómez (62)	Maternal serum during third trimester of pregnancy	o,p’-DDT, p,p’-DDT, p,p’-DDE	74 boys, followed at 0, 1, 3, 6 and 12 mo	Mexico	Significant positive association between p,p’-DDE and anopenile length/height. Negative association between mixture of DDT isomers and its metabolites and anopenile length/height.
Torres-Sanchez (76)	Maternal serum before and during pregnancy	p,p′‐DDE and p,p′‐DDT	37 boys (age 3, 6, 12 or 18 months)	Mexico	Significant negative association between anal position index (anoscrotal distance per coccyx-scrotal distance) and first trimester DDE levels.
Dalsager (77)	Maternal urine during gestation (gw 28)	pesticide metabolites 3-PBA, TCPY, 2,4-D and DAPs	420 boys examined 3 months after expected date of delivery	Denmark	2,4-D levels: Second tertile compared to the first tertile was associated with shorter anoscrotal and anopenile distance
**Phenols**					
Mammadov (78)	Cord serum	BPA	72 newborn boys	Cyprus	BPA level above the 90^th^ percentile was associated with significantly shorter anoscrotal distance.
Sunman (79)	Cord blood	BPA	100 newborns (4 had hypospadias, 3 cryptorchidism, 7 retractile testes)	Turkey	Anogenital distance/weight correlated significantly with BPA levels (only in univariate analysis)
Fisher (70)	Maternal serum during pregnancy	9 phenols	234	UK	NS
Arbuckle (80)	Maternal first trimester urine sample	BPA, Triclosan	198 newborn boys	Canada	NS
Huang (81)	Maternal urine collected during pregnancy	BPA, nonylphenol	86 newborn boys	Taiwan	NS
Lassen (82)	Maternal urine during pregnancy	Triclosan	245 examined 3 months after expected date of delivery	Denmark	Negative association between triclosan levels and anogenital distance (borderline significance)
Liu (83)	Maternal urine during pregnancy (third trimester)	BPA, 4-nonylphenol, 4-t-octylphenol.	137 newborn boys	China	NS
Sun (84)	Maternal urine collected during pregnancy	BPA	555 newborn boys, follow-up at 6 months (n=343) and at 12 months (n=320)	China	Maternal exposure to BPA was associated with shorter anoscrotal & anopenile distance of the son at 12 months. No dose-response relationship
**Phthalates**					
Huang (85)	Amniotic fluid	Five phthalate metabolites	33 newborn boys	Taiwan	NS
Sunman (79)	Cord blood	DEHP, MEHP	100 newborns (4 had hypospadias, 3 cryptorchidism, 7 retractile testes)	Turkey	DEHP levels showed negative association with anogenital index.
Fisher (70)	Maternal serum during pregnancy	16 phthalate metabolites	239	UK	NS
Adibi (86)	Maternal first trimester urine sample	8 phthalate metabolites	354 newborn boys	USA	MnBP and MEHP levels were negatively associated with anoscrotal distance
Arbuckle (80)	Maternal first trimester urine sample	11 phthalate metabolites	198 newborn boys	Canada	MnBP levels and molar sum of low molecular weight phthalate metabolites were positively associated with anopenile distance.
Barrett (87)	Maternal first trimester urine sample	9 phthalate metabolites	366 newborn boys	USA	Molar sum of DEHP metabolites, and levels of MEOHP and MEHHP were negatively associated with anoscrotal and anopenile distance in the lower stress group. In the lower stress group MECPP and MnBP levels were negatively associated with anoscrotal distance.
Bornehag (88)	Maternal first trimester urine	Ten phthalate metabolites	196 boys (mean age 21 months)	Sweden	Levels of oh-MMeOP and oxo-MMeOP and sum of DiNP metabolites were negatively associated with anoscrotal distance
Bustamante-Montes (89)	Maternal urine during pregnancy (third trimester)	4 phthalate metabolites	73 newborn boys	Mexico	Negative association between total phthalate levels and anopenile distance.
Huang (85)	Maternal urine during pregnancy	5 phthalate metabolites	33 newborn boys	Taiwan	NS
Jensen (90)	Maternal urine during pregnancy	12 phthalate metabolites	245 boys 3 months after the date of expected delivery	Denmark	NS
Martino-Andrade (91)	Maternal urine collected in each trimester	11 phthalate metabolites	168 newborn boys	USA	NS (tendency to negative association between anoscrotal and anopenile distance and DEHP metabolite levels in the first trimester)
Suzuki (92)	Maternal urine during pregnancy	seven phthalate metabolites	111 newborn boys	Japan	Negative association between anopenile distance/weight and MEHP level.
Swan (93)	Maternal urine during pregnancy	nine phthalate monoester metabolites	85 boys (median age 14 months)	USA	Levels of MEP, MBP, MBzP, MiBP and their summary phthalate score were negatively associated with anopenile distance/weight.
					Levels of MEP were also inversely associated with anoscrotal distance/weight.
Swan (94)	Maternal urine during pregnancy	nine phthalate monoester metabolites	106 boys aged 2-36 months (extension of study by Swan et al., 2005)	USA	Levels of MEP, MBP, MEHP, MEOHP and MEHHP were negatively associated with anopenile distance.
Swan (95)	First trimester urine sample	11 phthalate metabolites	366 newborn boys	USA	MEHP, MEOHP, MEHHP and sum of DEHP metabolite levels were significantly and negatively associated with anoscrotal or anopenile distance.
Wenzel (96)	Maternal urine from second trimester	8 phthalate metabolites	171 newborn boys	USA	Negative association between MEHP and anopenile distance. Positive association between molar sum of DBP metabolites or MiBP levels and anoscrotal distance.

NS, no statistically significant association

Only statistically significant findings are shown.

Many, but not all, studies listed in **Table 1** suggested negative associations between anoscrotal or anopenile distance and phthalate levels in maternal urine samples collected during pregnancy. A recent meta-analysis found that sum of di(2-ethylhexyl) phthalate (DEHP) metabolites in maternal urine was associated with a risk of short anoscrotal and anopenile distance in the son (97). In addition, monoethylhexyl phthalate (MEHP), mono(2-ethyl-5-hydroxyhexyl) phthalate (MEHHP), mono(2-ethyl-5-oxohexyl) phthalate (MEOHP) and mono(2-ethyl-5-carboxypentyl) phthalate(MECPP) levels (metabolites of DEHP) were associated with the risk of shortened anopenile and anoscrotal distance (97). Furthermore, monobutyl phthalate (MBP), monoethyl phthalate (MEP), and monoisobutyl phthalate (MiBP) levels were associated with the risk of shortened anopenile distance (97). A previously published systematic review included less studies than our review or the above-mentioned recent meta-analysis and it suggested moderate evidence for a negative association between DEHP and dibutylphthalate (DBP) exposure and anogenital distance in boys, and slight evidence for diisononyl phthalate (DiNP), butyl benzyl phthalate (BBP), diethyl phthalate (DEP) and diisobutyl phthalate (DiBP) (51).

In **Table 1**, three out of four studies suggested a negative association between PCB or PBDE exposure levels and anogenital distance. BPA/phenol levels were negatively associated with anogenital distance in less than half of the listed studies. Negative associations between pesticide exposure levels (different chemicals included) and anogenital distance in the boys were reported in less than half of the studies. For some chemical groups, only a few human studies have been published so far and it is difficult to draw any conclusions. Differences in results of the studies may be explained by variation in exposure levels, in timing of the sample collection, in matrices analyzed, in the age of the boys at examination, in other factors included in the statistical analysis (e.g., stress), and in metabolites/chemicals analyzed. It also has recently been suggested that human-rodent differences in results concerning associations between prenatal EDC exposure and anogenital distance could be due to species differences in regulation of fetal androgen production (98).

### 2.2 Cryptorchidism

Congenital cryptorchidism, i.e. undescended testis, is one of the most common congenital malformations in newborn boys and prevalences between 1 and 8 percentage have been reported in full term boys in prospective cohort studies (4). Testicular descent from the intra-abdominal position into the scrotum is usually completed by 35th GW [reviewed in (99)]. Proper androgen action is important especially for the last phase of testicular descent, the inguinoscrotal phase (100). Furthermore, the first phase of testicular descent is, at least in mice, dependent on insulin-like peptide 3 (INSL3), a hormone produced by Leydig cells, and estrogens have been shown to downregulate the expression of INSL3 gene (99, 101). Therefore, fetal exposure to environmental chemicals with anti-androgenic and estrogenic properties might be associated with cryptorchidism in boys.

For pesticides, several studies have been published, and nine out of 14 studies listed in **Table 2** suggested no significant association with the risk of cryptorchidism. All but two studies (one for each group) in **Table 2** found no significant association between PCB or phthalate exposure levels and the risk of cryptorchidism. Two out of five studies suggested that PBDE exposure levels are positively associated with the risk of cryptorchidism. For phenols, two out of five studies suggested positive association between BPA exposure levels and the risk of cryptorchidism. For dioxins, perfluorinated compounds, parabens, organotins and solvents, only a few studies have been published so far and it is difficult to draw any conclusions. In a study evaluating simultaneously the risk of cryptorchidism and levels of several congeners of different chemical groups, levels of four PBDEs and octachlorodibenzofuran (OCDF) were significantly higher in the group representing Danish cryptorchid boys when compared with controls (131).

**Table 2:** Case-control studies on the association between exposure to different classes of environmental EDCs (based on matrix measurements) and cryptorchidism in boys.

Reference	Matrix	Chemicals/congeners analysed	N of cases/controls	Country	Association between chemical levels and cryptorchidism
**Dioxins**					
Koskenniemi (102)	Boy’s adipose tissue	17 PCDD/Fs, total-TEq	30/29	Finland	Significant positive association with the risk of cryptorchidism (sum of 17 PCDD/Fs, total-TEq)
			14/9	Denmark	
Virtanen (103)	Placenta	17 PCDD/Fs, dioxin WHO-TEq, total-TEq	56/56	Finland	NS (sum of dioxins, dioxin WHO-TEq, total-TEq)
			39/129	Denmark	
**Flame retardants**					
Koskenniemi (102)	Boy’s adipose tissue	14 PBDEs	30/29	Finland	NS (sum of PBDEs)
			14/9	Denmark	
Goodyer (104)	Maternal hair (after pregnancy)	8 PBDEs	137/158	Canada	BDE-99, BDE-100 and BDE-154 levels were positively associated with the risk of cryptorchidism
Small (105)	Maternal serum before or after conception	PBB-153	9/450	USA	NS
Main (106)	Maternal breast milk	14 PBDEs	33/32	Finland	In Denmark PBDE levels were significantly higher in cases than in controls (sum of 7 most prevalent PBDEs)
			29/36	Denmark	
Main (106)	Placenta	14 PBDEs	56/56	Finland	NS
			39/129	Denmark	
**Organotins**					
Rantakokko (107)	Placenta	MBT, DBT, TBT, TPhT, sum of OTCs	56/56	Finland	Denmark: DBT: significant positive association with the risk of cryptorchidism.
			39/129	Denmark	Finland: Highest tertile of TBT and DBT: Significant negative association with the risk of cryptorchidism
**Parabens**					
Fisher (70)	Maternal serum during pregnancy	6 parabens	55/277	UK	NS
**PCBs**					
Hosie (108)	Adipose tissue	6 PCBs and their sum	18/30	Germany	NS
Koskenniemi (102)	Adipose tissue	37 PCBs	30/29	Finland	NS (sum of PCBs close to significant)
			14/9	Denmark	
Brucker-Davis (109)	Cord serum	7 PCBs and their sum	67/84	France	NS
Brucker-Davis (109)	Maternal breast milk	7 PCBs and their sum	56/69	France	Cases were more often in the highest exposure group (sum of PCBs)
Chevalier (110)	Maternal breast milk	PCB153	52/128	France	NS
Axelsson (111)	Maternal serum (first trimester)	PCB-153	163/161	Sweden	NS
McGlynn (112)	Maternal serum (third trimester)	11 PCBs and their sums	230/593	USA	NS
Virtanen (103)	Placenta	37 PCBs, PCB WHO-TEq	56/56	Finland	NS (sum of PCBs, PCB WHO-TEq)
			39/129	Denmark	
Mol (113)	Umbilical cord	sum of PCBs 138, 153 and 180	19 boys with a history of cryptorchidism + 1 testis torsion/176	Faroe Islands (Denmark)	NS
**Per-fluorinated compounds**					
Toft, Anand-Ivell (114, 115)	Amniotic fluid	PFOS	270/300	Denmark	NS
			146/190 (gw 13-16)		
Vesterholm Jensen (116)	Cord blood	PFOS, PFOA and their metabolites	78/78	Finland	NS
			29/30	Denmark	
**Pesticides**					
Hosie (108)	Boy’s adipose tissue	DDT and metabolites, toxaphene, HCH, chlorinated cyclodienes, chlorinated benzenes	18/30	Germany	Cryptorchid boys had higher levels of HCE and HCB
Brucker-Davis (109)	Cord serum	DDE	67/84	France	NS
Rouget (117)	Cord plasma	Chlordecone	17/310	Guadeloupe (French West Indies)	NS
Brucker-Davis (109)	Maternal breast milk	DDE	56/69	France	Cases tended to be more often in the highest exposure group (borderline significance)
Chevalier (110)	Maternal breast milk	DDE	52/128	France	NS
Damgaard (118)	Maternal breast milk	27 organochlorine pesticides	62/68	Finland & Denmark	Cases had significantly higher levels than controls (combined analysis of 8 most abundant pesticides)
Rouget (117)	Maternal plasma at delivery	Chlordecone	23/382	Guadeloupe (French West Indies)	NS
Axelsson (111)	Maternal serum (first trimester)	p,p’DDE, HCB	165/165	Sweden	NS
Bhatia (119)	Maternal serum during or after pregnancy	DDE, DDT	75/283	USA	NS
Longnecker (120)	Third trimester maternal serum	DDE	219/552	USA	NS
Pierik (121)	third trimester maternal serum	HCE, HCB, β-HCH, oxychlordane, dieldrin, p,p’-DDE, p,p’-DDT	219/564	USA	Risk of cryptorchidism was significantly increased only for β-HCH levels between 50^th^ and 90^th^ percentiles
Trabert (122)	Third trimester maternal serum	transchlordane, oxychlordane	217/557	USA	NS
Waliszewski (123)	Maternal serum postpartum	HCB, beta-HCH, pp’DDE, op’DDT, pp’DDT, sum of DDT	30/30	Mexico	No significant difference between groups in mean and median levels, but risk ratio of cryptorchidism above one for exposure to HCB, pp’DDE, op’DDT, pp’DDT, sum of DDT
Fratrić (124)	Maternal urine postpartum	organo-phosphate metabolite dimethyl phosphate	30/30	Serbia	NS
**Phenols**					
Komarowska (125)	Boy’s serum	BPA	98/57	Poland	Total and conjugated BPA levels were higher in cases
Fénichel (126)	Cord blood	unconjugated BPA	46/106	France	NS
Chevalier (110)	Cord blood	BPA	52/128	France	NS
Fisher (70)	Maternal serum during pregnancy	9 phenols	52/274	UK	BPA levels were positively associated with the risk of cryptorchidism
Chevrier (127)	Maternal urine during pregnancy	BPA, benzophenone 3, triclosan, 2,4-dichlorophenol, 2,5-dichlorophenol, methyl-, ethyl-, propyl- and butylparaben, sum of parabens	38/113	France	NS
**Phthalates**					
Anand-Ivell (115)	Amniotic fluid (g w 13-16)	DEHP and DiNP metabolites 7cx-MMeHP and 5cx-MEPP	146/190 (gw 13-16)	Denmark	NS
Jensen (128)	Second- trimester amniotic fluid	DEHP metabolite 5cx-MEPP, DiNP metabolite 7cx-MMeHP	270/300	Denmark	NS
Brucker-Davis (109)	Cord serum	DBP,	67/84,	France	NS
		mBP	36/49		
Brucker-Davis (109)	Maternal breast milk	DBP,	56/69,	France	NS
		mBP	31/40		
Chevalier (110)	Maternal breast milk	mBP	52/128	France	NS
Main (129)	Maternal breast milk	6 phthalate monoesters	62/68	Finland & Denmark	NS
Fisher (70)	Maternal serum during pregnancy	16 phthalate metabolites	55/279	UK	No consistent association
Swan (94)	Maternal urine during pregnancy	9 phthalate metabolites	12/107	USA	DEHP metabolite (especially MEHP) levels were associated positively with the probability of cryptorchidism
Chevrier (127)	Maternal urine during pregnancy	11 phthalate metabolites:	50/149	France	NS
		sum of low- molecular weight phthalates,			
		sum of 4 DEHP metabolites,			
		sum of high- molecular weight phthalates			
**Solvents**					
Warenbourg (130)	Maternal urine during pregnancy	Glycol ether metabolites MAA and PhAA	14/41	France	NS
**Combined exposures**					
Brucker-Davis (109)	Maternal breast milk	Composite score: DDE, sum of PCBs, mBP	31/40	France	All exposures: NS
			56/69		DDE + sum of PCBs: Cases tended to be more often in the highest exposure group (borderline significance)
Krysiak-Baltyn (131)	Maternal breast milk	PBDEs	29/36	Denmark	Only in Denmark:
		PBBs	33/32	Finland	-Higher levels in case group: PBDE 119, 85, 75, 138, OCDF
		phthalate metabolites			-Higher levels in control group: PCB 18, 51, 33, 49 and 52
		organochlorine pesticides			
		PCBs			
		dioxins			
		(106 chemicals included in the combined analysis)			

NS, no statistically significant association.

Only statistically significant findings are shown.

Bonde et al. studied associations between in utero or infant exposure to environmental EDCs and cryptorchidism in a meta-analysis (132). The analysis included studies based on chemical measurements of different biological matrices. No significant association was observed between exposure to environmental EDCs and cryptorchidism, when including eight studies in the analysis (132).

#### 2.2.1 Association Between EDC Exposure and Hormone Levels in Early Life

Some of the above mentioned studies on cryptorchidism or anogenital distance have suggested association between EDC exposure levels and reproductive hormone levels of boys in amniotic fluid, cord blood or in serum samples taken at 3 months of age (79, 83, 103, 106, 107, 110, 114, 115, 126, 128, 129). In Danish case-control studies on cryptorchidism, amniotic fluid DEHP and DiNP metabolite and PFOS levels associated positively with amniotic fluid testosterone (T) levels and negatively with amniotic fluid Insulin-like peptide 3 (INSL3) levels (114, 115, 128). In French case-control studies on cryptorchidism, cord blood levels of BPA correlated negatively with cord blood INSL3 levels (110) and unconjugated BPA levels correlated positively with cord blood T and inhibin B levels (126). Maternal breast milk levels of PCB153, DDE or mBP did not correlate with cord blood INSL3 or T levels (110). In the Chinese study on anogenital distance, maternal urine BPA levels showed negative associations with boys’ cord blood T levels and T/estradiol (E2) -ratio (83). In the Turkish study on anogenital distance in boys, cord blood levels of BPA, phthalates and reproductive hormones were studied (79). BPA levels were positively associated with E2 levels in cord blood, but no other significant associations between chemical and reproductive hormone levels were observed (79).

The Danish-Finnish cryptorchidism study evaluated associations between EDC levels in breast milk (106, 129) or in placenta (103, 106, 107) and boy’s reproductive hormone levels at 3 months of age. Breast milk phthalate metabolite levels showed positive associations with boys’ Sex hormone- binding globulin (SHBG) levels, LH levels, LH/Free T –ratio, and negative association with boys’ Free T levels (129). Breast milk PBDE levels also showed significant positive association with boys’ LH levels (106). No other significant associations between PBDE levels in breast milk or placenta and boys’ reproductive hormone levels at 3 months were observed (106). Placenta PCB WHO-TEq levels also showed significant positive association with boys’ LH levels (only in the Finnish subjects) (103), but no significant association between placenta polychlorinated dibenzo-p-dioxins and dibenzofurans (PCDD/F) WHO-TEq levels and boys’ reproductive hormone levels was observed. Associations between placenta organotin levels and boys’ reproductive hormone levels differed between countries; they showed negative associations with LH levels and FSH/Inhibin B –ratio, and positive associations with inhibin B levels in the Finnish data, but in the Danish data, organotin levels in placenta showed negative associations with T levels and T/E2 –ratio (107). These results suggest that EDC exposures may affect except fetal but also postnatal testicular function in boys.

### 2.3 Hypospadias

In hypospadias, penile development is disturbed so that the opening of urethra is situated on the ventral side of the penis, or in the scrotum or perineum (133). Hypospadias is due to failed fusion of penile urethra folds during embryonic weeks eleven to sixteen (134, 135). Penile development is dependent on androgens (134). Both genes and environment are thought to have a role in the etiology of hypospadias (136).

Four out of eight studies listed in **Table 3** have suggested a positive association between pesticide levels and risk of hypospadias. For PCBs and phthalates, none of the few studies suggested significant positive association with risk of hypospadias. Only a few studies have evaluated so far the association between exposure to PBDEs, perfluorinated compounds, and solvents and conclusions are difficult to draw.

**Table 3:** Case-control studies on the association between exposure to different classes of environmental EDCs (based on matrix measurements) and hypospadias in boys.

Reference	Matrix	Chemicals/congeners reported	N of cases/controls	Country	Association between chemical levels and hypospadias
**Flame retardants**					
Poon (137)	Maternal hair (after pregnancy)	8 PBDEs	152/64	Canada	Hypospadias was associated with higher maternal hair PBDE levels (total and congeners 28, 47, 99, 153 and 154)
Koren (138)					
Carmichael (139)	Maternal mid-pregnancy serum	5 PBDEs	20/28	USA	NS
Small (105)	Maternal serum before or after conception	PBB-153	5/454	USA	NS
**PCBs**					
Carmichael (139)	Maternal mid-pregnancy serum	9 PCBs	20/28	USA	NS
Giordano (140)	Maternal serum after pregnancy	4 PCBs (118,138,153 and 180) and their sum	37/21	Italy	NS
McGlynn (112)	Maternal serum (third trimester)	11 PCBs and their sums	201/593	USA	NS
Rignell-Hydbom (141)	Maternal serum from early pregnancy	PCB-153	229/229	Sweden	NS
**Perfluorinated compounds**					
Toft (114) Anand-Ivell (115)	Amniotic fluid	PFOS	75/300	Denmark	NS
			48/190 (limited to g w 13-16)		
**Pesticides**					
Shekharyadav (142)	Boy’s blood	HCH, aldrin, dieldrin, endosulfan alpha, endosulfan beta, DDT and DDE	80/120	India	Hypospadias was associated with higher levels of DDE and beta- and gamma-HCH
Bhatia (119)	Maternal serum during or after pregnancy	DDT, DDE	66/283	USA	NS
Carmichael (139)	Maternal mid-pregnancy serum	DDT, DDE, HCB	20/28	USA	NS
Giordano (140)	Maternal serum after pregnancy	DDE, HCB	37/21	Italy	Positive association between risk of hypospadias and HCB levels
Longnecker (120)	Third trimester maternal serum	DDE	199/552	USA	NS
Rignell-Hydbom (141)	Maternal serum from early pregnancy	p,p’-DDE, HCB	237/237	Sweden	HCB: Highest exposure quartile was associated with higher risk of hypospadias
					DDE: Tendency to higher risk, but no statistically significant association
Trabert (122)	Third trimester maternal serum	Trans-nonachlor, oxychlordane	197/557	USA	NS
Haraux (143)	Meconium	11 pesticides and metabolites	25/58	France	Presence of 2-methy-4-chlorophenoxyacetic acid (MCPA) and isoproturon in meconium was associated with the risk hypospadias
**Phthalates**					
Anand-Ivell (115)	Amniotic fluid (weeks 13-16)	DEHP metabolite 5cx-MEPP and DiNP metabolite 7cx-MMeHP	48/190	Denmark	NS
Jensen (128)	Second- trimester amniotic fluid	DEHP metabolite 5cx-MEPP, DiNP metabolite 7cx-MMeHP	75/300	Denmark	NS
Chevrier (127)	Maternal urine during pregnancy	11 phthalate metabolites: sum of low- molecular weight phthalates,	19/57	France	Significantly lower risk of hypospadias with the second tertile of urinary levels of low molecular weight phthalates
		sum of 4 DEHP metabolites,			
		sum of high- molecular weight phthalates			
**Solvents**					
Warembourg (130)	Maternal urine during pregnancy	Glycol ether metabolites methoxyacetic acid (MAA), phenoxyacetic acid (PhAA)	15/45	France	Highest tertile of MAA levels was associated with a higher risk of hypospadias
**Combined exposures**					
Rignell-Hydbom (141)	Maternal serum from early pregnancy	PCB-153, DDE, HCB	229/229	Sweden	NS

NS, no statistically significant association.

Only statistically significant findings are shown.

In the meta-analysis by Bonde et al, also associations between exposure to environmental EDCs and hypospadias was studied (132). Based on 18 risk estimates no significant association was found (132). No significant link was either found when studying association of hypospadias with specific exposures to DDE (degradation product of pesticide DDT) and PCBs (132).

Some studies evaluated cryptorchid and hypospadias cases in combination. In a Spanish study Arrebola et al. included 29 cases (16 with cryptorchidism, 12 with hypospadias, and one with both disorders) and 60 healthy controls (144). They assessed anti-androgenic activity of placenta samples using total effective xenobiotic burden of anti-androgens (TEXB-AA) as a biomarker, combined with a bioassay-directed fractionation protocol. They found a significant positive association between TEXB-AA levels in fraction 2 and occurrence of genital malformations (144). Another study from Spain compared placenta levels of 16 organochlorine pesticides and total effective xenoestrogen burden between a group of boys with cryptorchidism or hypospadias (n=36) and a group of matched control boys (n=109) (145). Cases had more often measurable level of estrogenicity due to xenoestrogens (TEXB-alpha fraction) in their placenta (145). In addition, presence of five pesticides (o,p′-DDT, p,p′-DDT, endosulfan-α, lindane, and mirex) in placenta were associated with an increased risk of birth defects (cryptorchidism or hypospadias) (145). In another study, Fernandez et al. compared placenta levels of BPA, 6 benzophenones and 4 parabens in boys with genital malformations (cryptorchidism or hypospadias, n=28) to those of control boys (n=51) (146). The third tertiles of BPA and propylparaben (PP) levels were associated with significantly increased risk of urogenital malformations, but cryptorchidism and hypospadias were not analyzed separately (146).

Also a study from the USA evaluated cryptorchid and hypospadias cases in combination. Maternal first trimester urinary phthalate metabolite (n=6) levels were not significantly associated with the risk of cryptorchidism or hypospadias (n=5 and n=3, respectively, analyzed together, and n of controls = 334) (147). In a study from Turkey, cord blood BPA, DEHP and MEHP levels were not statistically different in patients (14 out of 100 boys) with either hypospadias, cryptorchidism or retractile testis compared to control boys (79). Another study from the USA evaluated association between in utero exposure to polybrominated biphenyls (PBBs) and cryptorchidism and hypospadias separately and combined (n of all boys = 393) (105). No association was observed in the analyses (105). In the above mentioned metanalysis by Radke et al., the evidence for association between phthalate exposure and cryptorchidism or hypospadias was slight or indeterminate (51).

Small studies on risk factors of hypospadias or cryptorchidism may have limited power to find statistically significant differences. Especially hypospadias is less frequent malformation and therefore inclusion of cases may be challenging. However, some of the studies that remained negative included almost two hundred cases and thus, limited number of cases seems unlikely reason for their negative result. Differences in severity of cases, in exposure levels, in timing of the sample collection, in matrices and statistical analyses may also explain differences in results of different studies.

### 2.4 Semen Quality

Epidemiological studies have reported a global decline in semen quality, particularly in countries of Western origin. In 1992, Carlsen et al. reported a considerable global decline of mean sperm concentration from 113 million/mL in 1938 to 66 million/mL in 1991 (148). This finding is confirmed by later meta-analyses, including a systematic review and meta-regression analysis by Levine et al. in 2017, which showed a decline in sperm concentration globally at a rate of 0.70 million/mL/year from 1973 to 2011 (1). The decrease in sperm concentration and total sperm count was significant only among men in North America, Europe, Australia and New Zealand, which have a population of the European descent, but not in other regions (1). The cause of deteriorating semen quality is still unclear; however, some research supports the role of EDC exposure. Here, we review epidemiological studies that investigated the association between EDC exposure and results from standard semen analysis. We include only studies that reported chemical measurements in biological matrices.

#### 2.4.1 Early Life Exposure

##### 2.4.1.1 Phenols: Bisphenol A

Hart et al. studied associations between prenatal exposure to BPA and semen quality among Western Australian Pregnancy Cohort (Raine) Study men aged 20-22 years (149). A total of 284 men had maternal serum measured for BPA levels. Serum samples were collected at 18^th^ and 34^th^ week of gestation and pooled for the statistical analysis. Maternal serum BPA levels were positively associated with sperm concentration and progressive sperm motility, but not with other semen quality parameters, after adjustment for maternal smoking, duration of sexual abstinence and the presence of varicocele (149). This result did not support the link between BPA and poor semen quality. However, the BPA level was measured in the serum, and not in the urine, which is the standard method of assessment. In addition, BPA levels at the adult age were not measured, and therefore the BPA exposure in adulthood was not determined.

##### 2.4.1.2 Polychlorinated Biphenyls, dioxins

Some studies have reported an association between dioxins and PCBs and low semen quality (150, 151). Guo et al. reported that men born to mothers who had been exposed to PCBs and/or polychlorinated dibenzofurans (PCDFs) during pregnancy (n=12) had higher percentage of sperm with abnormal morphology and lower percentage of total or progressive motility as compared with men who were born to non-exposed women (n=23) (151). In an Italian study, 21 men who had prenatal exposure to dioxins due to a factory accident in Seveso in 1976, were observed to have lower sperm concentration, total sperm count, percentage of progressive sperm motility, and total motile sperm count than the 36 controls (150). This finding supports a link between prenatal exposure to PCBs and/or PCDFs and poor semen quality. In contrast, a study of 176 young men from a pregnancy cohort in Denmark showed that maternal serum ∑PCB and ∑DL-PCB levels collected at 30^th^ week of pregnancy were not correlated with semen quality of the sons (152).

##### 2.4.1.3 Phthalates

Hart et al. studied association between prenatal phthalate exposure and reproductive health in adulthood in the above-mentioned Raine study (153). The study showed that pooled maternal serum levels of monoisononyl phthalate (MiNP), sums of DEHP and DiNP metabolites and the sum of high molecular weight phthalates collected at 18 and 36 weeks of pregnancy were negatively associated with testicular volume of the sons in adulthood. Maternal serum MEP levels were negatively associated with semen volume and mono-carboxy-iso-octyl phthalate (MCiOP) levels were negatively associated with progressive sperm motility (153). Axelsson et al. analyzed association between maternal serum levels of DEHP- and DiNP metabolites during pregnancy and semen quality of the 112 sons (154). They reported that men who had MEHHP and MCiOP exposure levels in the highest tertile had lower semen volume than those of men in the lowest exposure tertile (154). The results of these studies suggested a potential role of prenatal exposure to phthalates in determination of semen quality.

The mechanism of the association between phthalate exposure and poor semen quality in men is unclear. Studies in animals, such as rodents, demonstrated that prenatal phthalate exposure, particularly during masculinization programming window, can disrupt fetal testis development and cause a reduced androgen production. This effect can result in a variety of male reproductive disorders postnatally (63, 155–159). Fetal testis xenograft into castrate male nude mice showed that serum testosterone did not differ between vehicle and DBP-exposed hosts (52). This finding suggested that human fetal testes exposure to DBP did not impair fetal testicular testosterone production as shown in animal studies (52). However, an increased amount of multinucleated germ cells were observed in the testes exposed to DBP, indicating an adverse effect on spermatogenesis (158). Some animal studies have shown that some phthalate metabolites can act as estrogen receptor agonists by binding to estrogen receptor α or β (160).

##### 2.4.1.4 Pesticides: DDT and Degradation Products

One case-control study showed that mothers of subfertile men had significantly higher serum p,p’-DDE levels than mothers of the fertile men, which indirectly suggest the link between prenatal exposure to p,p’-DDE and male infertility (161). However, maternal serum DDE levels were measured when the men were in adult age, not during pregnancy. A pregnancy cohort study in Denmark showed that maternal level of p,p’-DDE during pregnancy was not associated with sons’ semen quality (152).

##### 2.4.1.5 Perfluorinated Compounds

A Danish pregnancy cohort study showed a negative association between maternal serum PFOA level during pregnancy and adjusted sperm concentration and total sperm count of the sons at the young adult age (162). There was no significant association between maternal serum PFOS level and semen quality of the sons (162).

In summary, there is a limited number of studies on the association between prenatal exposure to EDCs and semen quality in adulthood. Some studies demonstrated a link between prenatal EDC exposures and poor semen quality, supporting the testicular dysgenesis syndrome (TDS) hypothesis, which stated that prenatal EDC exposure can interfere with fetal testicular development and function and may result in long-term reproductive health problems (11, 163). For EDCs with a long half-life, e.g., persistent organic pollutants (POPs), some studies use the concurrent measurement of EDCs in men or their mothers and semen quality, assuming that these EDC levels may reflect exposure since the fetal or infancy period. However, EDC exposures may have continued postnatally, and therefore, the timing of endocrine disrupting effects cannot be clearly identified.

The studies on the association between prenatal exposure to EDCs and semen quality are summarized in **Table 4**. Owing to a limited number of studies and inclusion of only few birth cohort studies, no conclusions can be drawn at the moment. More birth cohort studies are needed to better illustrate the role of prenatal EDC exposures in poor semen quality.

**Table 4:** Studies on early life endocrine disrupting chemical exposure and associations with semen quality.

EDC class	Reference	Matrix	Study design	Chemicals/congeners reported	N of subjects	Country	Association between chemical levels and semen quality
**Phenols: BPA**							
Hart 2018	(149)	Maternal serum	Cohort	Maternal serum for total BPA (free+ conjugated)	136 men (20-22 years of age)	Australia	Positive association between maternal serum BPA levels and sperm concentration and motility of the sons
				Serum FSH, LH			
**Dioxins**							
Mocarelli 2011	(150)	Serum	cohort	Maternal dioxin level at conception	39 men born to mothers who exposed to dioxin following the accident in Seveso, Italy (mean age, 22.5 y ± 2.2 y) *vs* 58 comparisons (mean age = 24.6 y ± 2.0)	Italy	21 breast-fed sons *vs* 36 breast-fed comparisons: lower
							- sperm concentration
							- total sperm count
							- progressive motility
							- total motile count
							Formula fed exposed *vs* formula-fed and breast-fed comparisons: no sperm related differences
**Phthalates**							
Hart 2018	(153)	Maternal serum (pooled at 18 and 34 GW)	Cohort	Maternal serum (pooled at 18 and 34 GW) for 32 phthalate metabolites	423 men (20-22 years of age)	Australia	Negative association between
							- antenatal serum MEP levels and seminal volume
					111 men who provided semen samples		- MCiOP level and sperm
							motility
Axelsson 2015	(154)	Maternal serum	Cross-sectional	Maternal serum for metabolites of DEHP and DiNP	112 adolescent males, aged 17.5-20.5 y	Sweden	Semen volume of the men with the highest tertile of MEHHP and MCiOP was lower than men with the lowest tertile.
**PCBs and pesticides (p,p’-DDE)**							
Vested 2014	(152)	Maternal serum	Birth cohort	Maternal serum for 6 PCBs and p,p’-DDE (pregnancy week 30)	176 men, aged 19-21 y	Denmark	No associations between maternal serum ∑PCBs, ∑DL-PCB, p,p’-DDE levels and semen quality of the sons
				Semen and blood sample from each son			
**Perfluorinated compounds**							
Vested 2013	(162)	Serum	Birth cohort	Maternal serum for PFOA and PFOS (pregnancy week 30)	169 men, aged 19-21 y	Denmark	Maternal serum PFOA levels had negative association with sperm concentration and TSC (only in adjusted models)
				Semen and blood sample from each son			Maternal serum PFOS: NS

NS, no statistically significant association.

Only statistically significant results are shown.

#### 2.4.2 Postnatal Exposure

There is some evidence to support a relationship between postnatal exposure to some endocrine disrupting chemicals and low semen quality. The studies are summarized in **Table 5**.

**Table 5:** Studies on postnatal endocrine disrupting chemical exposure and associations with semen quality.

EDC class	Reference	matrix	Study design	Chemicals/congeners reported	n of subjects	Country	Association between chemical levels and semen quality
**Phenols: BPA**							
Adoamnei 2018	(164)	Urine	Cross-sectional	BPA	215 university students	Spain	Negative association with sperm concentration and TSC
					(age range, 18–23 y)		
Caporossi 2020	(165)	Urine	Cross-sectional	BPA	155 male partners of subfertile couples, aged 40.5 y	Italy	Positive association between BPA level and semen volume
Ji 2018	(166)	Urine	Cross-sectional	BPA	500 fertile men, aged 18-55 y	China	Negative association with sperm concentration and sperm swing characteristics and positive association with sperm velocity ratios.
Kranvogl 2014	(167)	urine	Cross-sectional	BPA	136 male partners of infertile couples (mean age, 36.2 y)	Slovenia	No association between BPA and sperm concentration or sperm motility
Meeker 2010	(168)	Urine	Cross-sectional	BPA	190 men attending infertility clinic	USA	Negative association with sperm concentration, motility and morphology
					Mean age, 37 y		
Mendiola 2010	(169)	Urine	Cross-sectional	BPA	302 fertile men	USA	NS
					Mean age, 31.9 y		
Li 2011	(170)	Urine	Cohort	BPA	218 men with and without occupational BPA exposure	China	Negative association with sperm concentration, TSC and sperm motility
					(age from <25 to > 45y)		
Knez 2014	(171)	Urine	Cohort	BPA	149 male partners of couples undergoing IVF	Slovenia	Negative association with sperm concentration and TSC
					(mean age, 34 y)		
Lassen 2014	(172)	Urine	Cross-sectional	BPA	308 young men from general population	Denmark	Men in the highest quartile of BPA had significantly lower % progressive motile spermatozoa *vs* men in the lowest quartile
							No association with other semen parameters
Goldstone 2015	(173)	Urine	Cohort	BPA	418 male partners of couples trying to conceive	USA	NS
					(mean age, 31.7 y)		
Hu 2017	(174)	Urine	Cross-sectional	BPA	357 subfertile men	China	NS
					(mean age, 28.7 y)		Among obese men, negative association with sperm concentrations and total sperm counts
Omran 2018	(175)	Urine	Case-control	BPA	50 infertile men and 50 controls	Egypt	urinary BPA levels did not differ between infertile men and controls.
							All participants: urinary BPA levels: positive association with percentage of abnormal sperm morphology
							Negative association with progressive sperm motility and total sperm counts
Pollard 2019	(176)	Urine	Cross-sectional	BPA	161 men, aged 18-40 y with unknown subfertility	USA	Men with abnormal sperm tail morphology had higher geometric mean of BPA exposure than men with normal sperm morphology
Radwan 2018	(177)	Urine	Cross-sectional	BPA	315 men, aged <45 y, who had sperm concentration of ≥ 15 million/ml	Poland	Negative association with sperm motility
Vitku 2015	(178)	Plasma seminal fluid	Cross-sectional	BPA	174 men attending the fertility center	Czech Republic	Slightly infertile men had higher BPA levels in plasma and seminal plasma as compared with healthy men.
					Mean age, 36 y		Negative association between seminal BPA level (but not plasma BPA) and sperm concentration and TSC
Vitku 2016	(179)	Plasma and Seminal plasma	Cross-sectional	BPA	191 men attending infertility clinic	Czech Republic	Plasma BPA: NS
					Mean age, 36 y		Seminal plasma BPA: negative association with sperm concentration, TSC and normal sperm morphology.
**Phenols: Bisphenol S (BPS)**							
Ghayda 2019	(180)	Urine	Cross-sectional	BPS	158 men attending fertility center	USA	Men with detectable *vs* non-detectable BPS levels had lower semen volume, sperm concentrations, TSC and %morphologically normal sperm
					(age 18-56 y)		
**Parabens**							
Adoamnei 2018	(181)	Urine	Cross-sectional	BP	215 university students, aged 18-23 y	Spain	NS
				EP			
				MP			
				PP			
Jurewicz 2017	(56)	Urine	Cross-sectional	BP EP iBuP MP PP	315 men aged less than 45 y who attended the infertility clinic with normal semen concentration (15-300 million/mL)	Poland	Positive association with %sperm with abnormal morphology Negative association with %motility
Meeker 2011	(182)	Urine	Cross-sectional	Parabens	190 male partners attending infertility clinic who had semen analysis results	USA	NS
					Mean age, 36.7 y		
Smarr 2018	(183)	Urine	Cross-sectional	MP EP PP BP BzP HP 4-HB 3,4 DHB OH-MeP OH-EtP	501 male partners of couples planning to become pregnant Mean age = 31.8 y	USA	Negative association between EP, BP levels and sperm count Negative association between EP, MP levels and % sperm motility Negative association between BP level and CASA sperm motility parameters (average path velocity, straight-line velocity, curvilinear velocity, beat cross frequency, %straightness and %linearity) Positive association between OH-MeP level and %normal sperm (by WHO criteria) Positive association between OHEtP level and %normal sperm (by Strict and WHO criteria)
**Phthalates**							
Albert 2018	(184)	Urine	Cross-sectional	Phthalate metabolites	153 healthy men, aged 18-41 y	Canada	NS
Axelsson 2015	(185)	Urine	Cross-sectional	10 phthalate metabolites	314 men from general population, aged 17-20 y	Sweden	Negative association between all the DEHP metabolites (MEHP, MECPP, MEOHP, MEHHP, MBP) and progressive sperm motility
							Positive association of MCiOP, % MEHP with semen volume
Chang 2017	(186)	Urine and seminal fluid	Cross-sectional	Phthalate metabolites	253 male partners of subfertile couples	Taiwan	*Urinary phthalate metabolites*
							Negative associations between:
					37 male partners of fertile couples		- MBzP, MEHP, MEHPX and sperm concentration
					Mean age: 33 y		- MBzP, MEHP and sperm motility
							*Seminal phthalate metabolites*
							Negative association between
							- MEHP and mono-2-ethyl-5-hydroxyhexyl phthalates and sperm concentration
							- MEP, DEHP metabolites and sperm motility
							- MEP and morphologically normal sperm
Chen 2017	(187)	Urine	Cohort	Phthalate metabolites	796 male students who moved to a different university campuses (median age: 20 y)	China	Negative associations between
							- mEP and sperm concentration
							- mEP, MnBP, MCPP, ∑LMWP and sperm motility
							- MnOP, MEHP, ∑HMWP and morphologically normal sperm
							Positive associations between
							- miBP, MEHP and semen volume
							- MnOP and progressive motility
							- MBzP and sperm with normal morphology
							Levels of the phthalate metabolites, except MEHP, decreased, while semen volume and morphologically normal sperm increased after relocation.
Joensen 2012	(188)	Urinary phthalate metabolites	Cross-sectional	14 phthalate metabolites	881 men	Denmark	Men with the highest quartile of %MiNP had higher semen volume and % progressive motility *vs* lowest quartile
					Median age 19.1 y		
Jurewicz 2013	(189)	Urinary phthalate metabolites	Cross-sectional	mono(2-ethyl-5OH-MEHP), MEHP, DEP, MEP, BBzP, MBzP, DINP, MINP, DBP, MBP	269 men attending infertility clinic (sperm concentration ≥ 15 M/mL)	Poland	5OHMEHP, MEHP and MINP: Negative association with sperm motility
				Men’s serum FSH, TT and E2			
Kranvogl 2014	(167)	Urinary phthalates		9 urinary phthalate monoesters	136 male partners of infertile couples (mean age, 36.2 y)	Slovenia	MEHP, DMP, DBP, DEHP, MEOHP and sum DEHP levels were negatively associated with sperm concentrations.
							MEHP, DBP, MEOHP, sum DEHP levels were negatively associated with sperm motility.
Han 2014	(190)	Urinary phthalate metabolites	Cross-sectional	Urinary levels of MBP	232 men from 1 reproductive center	China	Weak association between urinary MBP levels and sperm concentration; men with MBP levels above median were 1.97 times more likely to have sperm concentration below the reference value.
				MEP	Mean age, 32 y		
				MEHP			
				MBzP			
				PA			
				Total PA			
				Semen analysis			
Pant 2008	(191)	Seminal phthalate metabolites	Cross-sectional	Seminal levels of DEP, DEHP, DBP, DMP and DOP	300 healthy men, aged 21-40 y	India	Negative association between DEP, DBP, DEHP levels and sperm concentration
							Negative association between DBP, DEHP and sperm motility
							Positive association between DEHP level and % abnormal sperm morphology
Pant 2011	(192)	Seminal fluid	Cross-sectional	DEHP and DBP	180 healthy men, aged 21-40 y	India	Negative association between DBP, DEHP levels and sperm motility
Pant 2014	(193)	Seminal fluid	Cross-sectional	DEHP	60 male partners of couples attending the andrology laboratory	India	Negative associations between DEHP and sperm motility, sperm concentration and normal morphology
				DBP	Age 21-40 y		
				DEP			
Liu 2012	(194)	Urine	Cross-sectional	6 urinary phthalate metabolites	97 men attended fertility clinic	China	NS
				(MMP, MEP, MBP, MBzP, MEHP and MEOHP)	(median age, 31.5 y)		
Pan 2015	(195)	Urine	Cross-sectional	14 phthalate metabolites	1066 men	China	Negative association between MBP, MiBP and % morphologically normal sperm
					(median age, 29 y)		Negative association between %MEHP and %progressive sperm motility
Smarr 2018	(196)	Seminal fluid	Cross-sectional	phthalate metabolites	339 male partners of couples discontinuing contraception to become pregnant, mean age 31.8 years	USA	Negative associations between mEP, mBP, miBP, mBzP and semen volume
Thurston 2016	(197)	Urine	Cross-sectional study	9 phthalate metabolites	420 partners of pregnant women Mean age, 32 y	USA	No association between DEHP, DBP, DEP, DOP levels and semen quality.
							Negative association between MBzP level and sperm motility
Wang 2016	(198)	Seminal fluid	Cross-sectional	8 phthalate metabolites	Male partners of subfertile couples	China	Negative associations between MBP, MEHP, MEHHP, MEOHP and semen volume
					Semen samples (n = 687) Blood samples (n = 342)		Positive associations between MBzP and abnormal sperm heads and tails.
Specht 2014	(199)	Urinary Secondary oxidized metabolites of DEHP and DiNP	Cross-sectional	5OH-MEHP oxo-MEHP 5cx-MEPP 7OH-MMeOP	589 male partners of pregnant women	Greenland Poland Ukraine	Negative associations between semen volume and proxy-MEHP, 5OH-MEHP and 7OH-MMeOP
				7oxo-MMeOP 7cx-MMeOP			Negative associations between TSC and proxy-MEHP and 5OH-MEHP
**Pesticides: Organophosphates**							
Miranda-Cantreras 2013	(200)	Erythrocyte acetylcholinesterase (AChE) and plasma butyrylcholinesterase activity	Cross-sectional	Erythrocyte acetylcholinesterase (AChE) and plasma butyrylcholinesterase activity	35 healthy farm male workers (unexpected group) and 64 male agricultural workers (exposed group)	Venezuela	No association between erythrocyte acetylcholinesterase (AChE) and plasma butyrylcholinesterase and semen quality
Melgarejo 2015	(201)	Urinary levels of 6 DAP metabolites	Cross-sectional	6 urinary DAP metabolites (organophosphate metabolites)	116 men, 25-38 years old	Spain	Negative correlation between urinary DMP levels and % sperm motility and morphologically normal sperm
					(median age, 35.1 y)		Negative association between urinary levels of DMP, DMTP, DMDTP, DAPs and sperm concentration and TSC
Perry 2011	(202)	Urine	Case control	6 DAPs	94 cases and 95 controls	China	Cases had higher urinary DMP levels *vs* controls
					Cases had higher sperm concentration and motility		
					Mean age, 26 y		
**Pesticides: Pyrethroids**							
Meeker 2008	(203)	Urine	Cross-sectional	Pyrethroid metabolites: 3-PBA	207 men	USA	Men in the highest 3-PBA quartile had lower sperm concentration than men with 3PBA of less than median.
				CDCCA	Mean age, 36 y		
				TDCCA			
Ji 2011	(204)	Urine	Cross-sectional	3-PBA	240 men from infertility clinic	China	Negative association between 3-PBA level and sperm concentration
Imai 2014	(205)	Urine	Cross-sectional	3-PBA	323 university students	Japan	NS
Radwan 2014	(206)	Urine	Cross-sectional	Pyrethroid metabolites: 3-PBA	334 men attended infertility clinic	Poland	Positive association between pyrethroid metabolite levels and %sperm with abnormal morphology
				CDCCA	Mean age: 32.2 y		Negative association between DBCA and curvilinear velocity and linearity
				TDCCA			
				DBCA			
Xia 2008	(207)	Urine	Cross-sectional	3-PBA	376 men with nonobstructive infertility	China	Men who had urinary creatinine-adjusted 3-PBA level in the 4^th^ quartile had higher risk of having sperm concentration < 20 million/mL
					Mean age 30.4 y		
**Pesticides: Organochlorines**							
Abdelouahab 2011	(208)	Serum	Cross-sectional	p-p′ DDE	52 men from a fertility clinic. age 25 – 50y	Canada	NS
Abou Ghayda 2020	(209)	Serum at the age of 8-9 y	Cohort study	HCB	152 young men provided semen samples	Russia	Negative association between semen volume and HCB and βHCH
				HCH			
				p,p’-DDE			
Aneck-Hahn 2007	(210)	Plasma	Cross-sectional	p,p’-DDE	311 men residing in the endemic malaria area	South Africa	Negative association between p,p’-DDE level and semen volume and mean CASA motility
					Mean age 23 y		
Ayotte 2001	(211)	Serum	Cross-sectional	p,p’-DDE	24 young men	Mexico	Negative association between serum p,p’-DDE level and semen volume and TSC
					Mean age 21 y		
De Jager 2006	(212)	Plasma	Cross-sectional	p,p’-DDE	116 men residing in the area of DDT use	Mexico	Negative association between plasma p,p’-DDE and percentage of sperm motility
					Mean age 27 y		
Hauser 2003	(213)	Serum	Cross-sectional	p, p’-DDE	212 male partners of subfertile couples	USA	NS
					Mean age 36 y		
Mumford 2015	(214)	Serum	Cross-sectional	9 organochlorine pesticides	501 male partners of couples trying to conceive	USA	Highest quartiles of some pesticides were associated with higher sperm concentration, total sperm count and sperm motility when compared to the lowest quartile.
					Mean age = 31.8 y		
Pant 2014	(215)	Seminal plasma	Cross-sectional	P,p’-DDE	193 infertile men	India	Men in the highest quartile of lindane or p,p-DDE had lower sperm concentration and motility.
				lindane	85 fertile men		
					Mean age, 28 y		
Specht 2015	(216)	Serum	Cross-sectional	HCB	589 fertile men	Greenland	Negative association between HCB and semen volume (only men in Greenland)
					Median age:	Poland	
					Greenland, 30.6 y	Ukraine	
					Poland, 29.6 y		
					Ukraine, 25 y		
**Perfluorinated Compounds (PFCs)**							
Louis 2015	(217)	Serum	Cross-sectional	7 PFCs	501 male partners of couples planning pregnancy	USA	Positive association between PFNA level and % morphologically normal sperm by Strict criteria
					Mean age ± SD, 31.8 ± 4.9 y		(NS by WHO criteria)
							NS (semen volume, sperm concentration, TSC, %motility)
Joensen 2009	(218)	Serum	Cross-sectional	10 PFAAs	105 men from general population	Denmark	Men with high levels of combined PFOS and PFOA had lower number of morphologically normal sperm than men with low levels of PFOS and PFOA.
					Median age 19 y		
Lewis 2015	(219)	Serum	Cross-sectional	4 PFASs	857 men from general population	USA	NS with T
					Age 12-80 y		
Petersen 2018	(220)	Serum PFASs	Cross-sectional	Serum PCBs, PFASs	263 men, aged 24-26 y	Faroe island	NS
Joensen 2013	(221)	Serum PFCs	Cross-sectional	14 PFCs	247 healthy men from general population	Denmark	PFHpS had negative association with progressive motility
					Median age = 19 y		
Raymer 2012	(222)	Plasma, seminal fluid	Cross-sectional	PFOS, PFOA	256 men came for fertility assessment	USA	NS
					Median age = 41 y		
Toft 2012	(223)	Serum	Cross-sectional	4 PFCs	588 partners of pregnant women Median age:	Greenland Poland Ukraine	Combined 3 countries: - Men who had PFOS or PFHxS level in the 3^rd^ tertile had lower % morphologically normal sperm than men in the 1^st^ tertile
					Greenland, 31.3 y	Ukraine	- Men who had PFOA level in the 3^rd^ tertile had higher % sperm motility
					Poland, 29.6 y		
					Ukraine, 26.2 y		
**PCBs and dioxins**								
Abdelouahab 2011	(208)	Serum	Cross-sectional	Sum of PCB-153, PCB-180, PCB-138	52 men from a fertility clinic. Age 25 – 50y	Canada	NS
Dallinga 2002	(224)	Serum and semen	Cross-sectional	PCB 118, 138, 153, 180,	65 men from infertility clinic	The Netherlands	Among men with good semen quality:
					Mean age:		Negative association between serum ∑PCB and TSC and progressive motile sperm
					Male factor subfertility group: 34.5 y		
					Female factor subfertility group: 36.7 y		
Hauser 2003	(213)	Serum	Cross-sectional	57 PCB congeners	212 male partners of subfertile couples	USA	Negative association between PCB-138 level and % sperm motility and % morphologically normal sperm
					Mean age 36 y		
Minguez-Alarcon 2017	(225)	Serum	Cohort (peripubertal exposure)	Serum PCBs dioxins, furans, PCBs, (age 8-9 y) Semen samples (age 18-19 y)	Healthy boys, aged 8–9 y (n=516) and 18–19 y (n=133)	Russia	Serum TCDD and PCDD TEQs: Negative association with sperm concentration, TSC, total motile sperm count
							Serum PCBs, furans and total TEQs: no association
Mumford 2015	(214)	Serum	Cross-sectional	36 PCB congeners	501 male partners of couples trying to conceive	USA	Highest quartiles of some congeners were associated with higher volume, total sperm count and sperm motility when compared to the lowest quartile
					Mean age = 31.8 y		
Paul 2017	(226)	Serum	Cross-sectional	DL-PCBs	Men, aged 30–55 y, from subfertile couples	Spain	*Men with normal semen quality:* negative associations between
					- low semen quality (n = 24)		- PCB-118 and semen volume
					- normal semen quality (n = 26)		- PCB-189 and progressive motility
							positive associations:
							- PCB-77, -123, total nonortho PCBs
							(sperm with normal morphology)
							*Men with low semen quality:*
							positive associations between
							- PCB-118, mono-ortho PCBs, PDLPCBs and semen volume
							- PCB-77, PCB-81 and morphologically normal sperm
Petersen 2015	(227)	Serum	Cross-sectional	PCB28,105,118,156, 52,101,153,138, 180	266 fertile men	Faroe Island	NS
					Median age, 34.8 y		
Petersen 2018	(220)	Serum	Cross-sectional	PCBs	263 men, aged 24–26 years	Faroe Island	NS
Richthoff 2003	(228)	Serum	Cross-sectional	PCB 153	305 young men from general population, aged 18-21 y	Sweden	Negative association between PCB 153 level and %sperm motility by CASA
Rignell-Hydbom 2004	(229)	Serum	Cross-sectional	PCB-153	195 fishermen, aged 24-65 y	Sweden	Lower sperm motility in men with the highest PCB-153 quintile as compared with men in the lowest quintile
Rignell-Hydbom 2005	(230)	Serum	Cross-sectional	PCB-153	176 fishermen	Sweden	NS
					Mean age: 47 y		
Rozati 2002	(231)	Semen	Cross-sectional	PCBs	21 infertile, mean age 33.7 y	India	Negative association between PCBs and total progressive motility
					32 controls, mean age 32.5 y		
Spano 2005	(232)	Serum	Cross-sectional	PCB-153	707 men	Greenland	NS
					Mean age:	Sweden	
					Inuit men 31.1 y	Ukraine Poland	
					Swedish fishermen 47.1 y		
					Warsaw men 30.3 y		
					Kharkiv men 26.6 y		
Toft 2006	(233)	Serum	Cross-sectional	CB-153 and p,p’-DDE	Men, aged > 18 y from All regions in Greenland (n = 194)	Greenland	Negative association between CB-153 level and sperm motility.
					Fishermen from Sweden (n = 185)	Sweden	No association between CB-153 and sperm concentration or %morphologically normal sperm
					Residents of Kharkiv in Ukraine (n= 195)	Ukraine	
					Residents of Warsaw in Poland (n = 189)	Poland	
Vitku 2016	(179)	Plasma and Seminal plasma	Cross-sectional	6 PCB congeners (PCB 28, 101, 118, 138, 153, 180)	191 men attended infertility clinic	Czech	∑PCBs (PCB 180, -153, -118 and -138) had positive association with sperm concentration and TSC
					Mean age 35 y		
**Flame retardants**							
Albert 2018	(184)	Hair	Cross-sectional	8 PBDE congeners	153 healthy men, aged 18-41 y	Canada	NS (tendency to negative association with sperm concentration and sperm motility)
Yu 2018	(234)	Seminal fluid	Cross-sectional	PBDEs	Cases: men aged 20–50 y residing at an e-waste dismantling workshop (n=32)	China	TSC, progressive motility, and total motile sperm were lower in cases than in controls.
					Controls: men aged 24–46 y (n=25)		Negative associations between seminal BDE-47 and sperm concentration and TSC
Abdelouahab 2011	(208)	Serum	Cross-sectional	BDE-47, BDE-99, BDE-100, BDE-153	52 men from a fertility clinic. Age 25 – 50y	Canada	Negative association with sperm motility
Akutsu 2008	(235)	Serum	Cross-sectional	29 PBDE congeners	10 men, aged 18 – 21 y	Japan	HxBDE-153 showed significant negative association with sperm concentration
Mumford 2015	(214)	Serum	Cross-sectional	10 PBDE congeners and PBB-153	501 male partners of couples trying to conceive	USA	Highest quartiles of some congeners were associated with lower sperm motility and higher sperm concentration and % of abnormal morphology when compared to the lowest quartile
					Mean age = 31.8 y		
Toft 2014	(236)	Serum	Cross-sectional	BDE-28, 47, 99,100, 153, 154 and 183, and BB-153	299 partners of pregnant women	Greenland, Poland and Ukraine	BDE-47 and BDE 153: NS (no consistent associations across countries)
					Median age:		
					Greenland, 32.1 y		
					Poland, 29.6 y		
					Ukraine, 26.1 y		

Only statistically significant findings have been shown.

Only studies reporting standard semen quality parameters are included.

NS, no statistically significant association.

##### 2.4.2.1 Phenols: Bisphenol A

To date, ten cross-sectional, one case-control and four cohort studies have evaluated the role of BPA exposure in semen quality and they have shown mixed results. All of the studies measured BPA in urine samples, except one study in which plasma and semen samples were analyzed for BPA (179). Most studies showed a negative association between urinary BPA level and sperm concentration and/or total sperm count (164, 166–168, 170, 174, 179). A negative association between seminal BPA, but not plasma BPA levels, and sperm concentration, total sperm count and percentage of morphologically normal sperm was found in one study (179). Urinary BPA levels were negatively associated with sperm motility in some studies (170, 177).

In summary, current evidence supports the link between BPA exposure in adulthood and poor semen quality, particularly low sperm concentration, total sperm count and sperm motility.

##### 2.4.2.2 Flame Retardants

Several studies have evaluated associations between PBDE levels in serum, hair or seminal fluid and semen quality. Most of them suggested negative associations with sperm concentration or sperm motility (184, 208, 214, 234, 235). One study including men from three countries found no consistent associations across countries (236).

##### 2.4.2.3 Phthalates

Evidence supports the link between phthalate exposure in adulthood and poor semen quality. A number of studies have shown negative associations of phthalate metabolite levels with semen quality, particularly sperm concentration (167, 186, 191, 193) and sperm motility (167, 186, 187, 189, 191, 192, 195, 197), although two studies showed a positive association between levels of some phthalate metabolites and sperm motility (187, 188). Two studies showed a negative association of phthalate metabolite levels with semen volume (196, 198, 199) and four studies demonstrated a negative association with percentage of morphologically normal sperm (186, 193, 195, 198). Three studies did not show any significant association with semen quality (184, 190, 194).

##### 2.4.2.4 Organochlorine Pesticides

Most studies on the association between p,p’-DDE levels and semen quality were conducted in the early 2000s. To date, evidence has supported an association between serum DDT or DDE levels and poor semen quality, particularly reduced sperm motility (210, 212, 215, 237, 238). Some studies also reported an association with low sperm concentration (210, 211), abnormal morphology (210) and low semen volume (210, 211). However, some studies did not show an association with semen quality (161, 208, 213, 229). One study reported positive associations between semen quality and serum levels organochlorine pesticides (214). Another study did not find significant associations between peripubertal serum p,p’-DDE levels and any semen parameters in adult men (209). The role of peripubertal p,p’-DDE exposure on semen quality needs to be studied further.

Studies on the association with semen quality and levels of other organochlorine pesticides, including lindane and hexachlorobenzene, are summarized in **Table 5**.

##### 2.4.2.5 Other Pesticides

In epidemiological studies, organophosphate exposure is usually assessed by detecting decreased blood, erythrocyte or plasma cholinesterase activity or detecting metabolites of organophosphates, for example dialkylphosphates (DAPs), in urine samples (239, 240). Possible relationship between organophosphate exposure and low semen quality has been shown (200–202, 241), however the number of studies are limited. Three studies showed a negative association between the level of 3-phenoxybenzoic acid (3-PBA), which is a metabolite of pyrethroids, and sperm concentration (203, 204, 207). However, one study did not report such a finding (205). One study showed that higher pyrethroid metabolite levels were associated with higher percentage of sperm with abnormal morphology, lower sperm concentration, and increasing rate of abnormal computer-assisted semen analysis (CASA) parameters, including lower curvilinear velocity and linearity (206).

##### 2.4.2.6 Parabens

Four cross-sectional studies investigated relationship between urinary paraben levels and semen quality and showed mixed results (56, 181–183). One study showed a significant positive association between urinary level of butylparaben (BP) and percentage of morphologically abnormal sperm and a negative association with percentage of sperm motility (56). Another study showed that urinary ethyl paraben (EP) and BP levels were negatively associated with sperm concentrations and urinary BP, EP and methyl paraben (MP) levels were negatively associated with percentage of sperm motility parameters by CASA. Levels of hydroxylated metabolites methyl-protocatechuic acid (OH-Me)P and ethylprotocatechuic acid (OH-EtP) were positively associated with percentage of morphologically normal sperm (183). However, two studies did not show any significant associations between paraben level and semen quality (181, 182). To summarize, there is limited amount of evidence suggesting a link between paraben exposure and semen quality.

##### 2.4.2.7 Perfluorinated Compounds

To date, four cross-sectional studies – two from Denmark, one from Faroe Island, and one from the USA – have examined the relationship between PFC exposure in adulthood and semen quality. Three studies did not find any significant associations between serum PFC levels and semen quality (220–222). Only one study from Denmark showed lower percentage of morphologically normal sperm in men who had high combined PFOA and PFOS levels as compared with those who had low levels (218).

##### 2.4.2.8 Polychlorinated Biphenyls

Several cross-sectional studies have demonstrated a link between PCB exposure, particularly PCB-153, in adulthood, and low semen quality (213, 224, 226, 228, 229, 231, 233, 242, 243), particularly low sperm motility (213, 224, 226, 228, 229, 231, 233). In contrast, one Chinese study showed an association with higher progressive sperm motility (238). In addition, this study also showed a positive association between sum of seminal dioxin-like PCB levels and semen volume, a negative association between seminal PCB-66, PCB-105 and sperm concentration, and a positive association between seminal PCB-44 and sperm concentration (238). A study on male partners of couples trying to conceive also reported positive associations between semen quality and serum levels of some PCB congeners (214). Whereas a study on men from a fertility clinic found no significant association between semen quality and serum PCB levels (208).

### 2.5 Reproductive Hormone Levels

#### 2.5.1 Early Life Exposure

A limited number of studies have investigated the association between prenatal exposure to EDCs and reproductive hormone levels in adult men. These studies are summarized in **Table 6**.

**Table 6:** Summary of studies that evaluated early life EDC exposure and adult reproductive hormone levels.

EDC class	Reference	Matrix	Study design	Chemicals/congeners reported	N of subjects	Country	Association between chemical levels and reproductive hormone levels
**Phenols: BPA**							
Hart 2018	(149)	Maternal serum	Cohort	Maternal serum for total BPA (free+ conjugated)	243 men (20-22 years of age)	Australia	No association between BPA and FSH, LH, inhibin B, T, LH:T, FSH:inhB, estradiol or estrone
**Phthalates**							
Axelsson 2015	(154)	Maternal serum	Cross-sectional	Maternal serum (mean 12 weeks of gestation) for metabolites of DEHP and DiNP	112 adolescent males, aged 17.5-20.5 y	Sweden	Highest tertile of MCiOP had higher FSH *vs* lowest tertile
							MCiOP and MOiNP: positively associated with FSH
							MHiNP and MOiNP: positively associated with LH
Hart 2018	(153)	Maternal serum	Cohort	Maternal serum (pooled at 18 and 34 GW) for 32 phthalate metabolites	Men in the birth cohort study (185 men had serum hormone measured and had maternal phthalate results), aged 20-22 y	Australia	TT at the age of 20-22 y: positively associated with MEHP, MiNP, ∑DEHPm, ∑DiNPm, ∑HMW phth.m and ∑DEHPm + DiNPm (adjusted for BMI)
				Serum for hormones of sons			NS (when adjusted for multiple comparisons)
							Positive association between MiNP level and FSH (adjusted for BMI)
							Negative association between MEHP level and LH:T ratio (adjusted for BMI)
							Positive association between ∑DEHPm and LH levels
							No association between prenatal phthalate metabolite levels and adult male serum inhibin B, E1 or E2 levels
**Dioxins**							
Mocarelli 2011	(150)	Maternal serum	Cohort	Maternal serum TCDD	39 men born to mothers who exposed to dioxin following the accident in Seveso, Italy (mean age, 22.5 y) *vs* 58 comparisons (mean age = 24.6 y)	Italy	Higher FSH and lower inhibin B in the breast-fed exposed group *vs* breast-fed comparisons
							Higher FSH and lower inhibin B in the breast-fed exposed group *vs* formula-fed exposed group
**Perfluorinated compounds**							
Vested 2013	(162)	Maternal serum	Birth cohort	Maternal serum for PFOA and PFOS (pregnancy week 30)	169 men, aged 19-21 y	Denmark	Positive association between maternal serum PFOA levels and FSH and LH levels in men.
**PCBs and p,p’-DDE**							
Vested 2014	(152)	Maternal serum	Birth cohort	Maternal serum for 6 PCBs and p,p’-DDE (at pregnancy week 30)	176 men at the age of 19-21 y	Denmark	NS

NS, no statistically significant association.

Only statistically significant findings are shown.

##### 2.5.1.1 Phenols: Bisphenol A

To date, there is no evidence supporting the relationship between maternal BPA exposure and reproductive hormone levels of the sons at the adult age. The pregnancy cohort in Western Australia ‘Raine study’ found no association of maternal serum BPA with hypothalamic-pituitary-gonadal (HPG) hormone levels of the sons (149).

##### 2.5.1.2 Phthalates

The Australian Raine study found that serum total testosterone levels of the sons at a young adult age were positively associated with maternal serum levels of serum phthalate metabolites during pregnancy, including MEHP, MiNP, the sum of DEHP and DiNP metabolites, and the sum of high molecular weight phthalates after adjustment for BMI (153). In addition, there was a positive association between maternal serum MiNP levels and FSH levels of the men and between maternal serum DEHP levels and serum LH levels of the men. A negative association between maternal MEHP level and serum LH to testosterone ratio in adult men was also observed. No association between maternal levels of phthalate metabolites during pregnancy and serum inhibin B or estradiol levels in adult men was found (153).

A study in 112 males, aged 17.5-20.5 years, and paired maternal serum samples collected at a mean of 12 weeks of gestation in Sweden demonstrated positive associations between maternal serum levels of MCiOP and mono-(oxo-iso-nonyl) phthalate (MOiNP) and FSH levels of the sons, and between maternal serum levels of two DiNP metabolites [mono-hydroxy-iso-nonyl phthalate (MHiNP) and MOiNP] and LH levels of the sons (154). DEHP metabolite levels in maternal serum were positively associated with total and free testosterone levels of the sons (154).

Results from these two studies suggested the potential long-term effects of prenatal phthalate exposure on the hypothalamic-pituitary-gonadal axis. However, more studies are needed to corroborate or refute these findings.

##### 2.5.1.3 Dioxins

Mocarelli et al. studied reproductive hormone levels of sons born to mothers who were exposed to dioxins during pregnancy due to an accident in Seveso, Italy, and compared them with hormone levels of sons born to mothers who had background exposure. Among breastfed group, 21 sons with maternal dioxin exposure had higher FSH and lower inhibin B levels than 36 sons with maternal background exposure (150). Among the maternal dioxin exposure group, breastfed sons (n=21) had higher FSH and lower inhibin B levels than formula-fed sons (n=18) (150). Among breastfed group, sons born to the exposed mothers had lower semen quality than sons born to the non-exposed mothers. These results suggest that in-utero and/or neonatal exposure to dioxins have a role in germ cell defects.

##### 2.5.1.4 PCBs, Pesticides (p,p′-DDE), and Perfluorinated Compounds

A birth-cohort study in Denmark showed that maternal serum PFOA level at 30^th^ week of pregnancy was positively associated with serum FSH and LH level of the sons at the age of 19-21 years (162). There was no significant association between maternal serum PFOS, PCBs, p,p’-DDE levels and serum levels of FSH, LH, testosterone, inhibin B, estradiol or SHBG in the adult sons (152, 162).

#### 2.5.2 Postnatal Exposure

Several studies have investigated the HPG axis hormone levels in adult men in relation to EDC exposure. Many studies examined the association of EDC exposure with testosterone levels. Some studies also evaluated pituitary FSH and LH levels. Only a small number of studies evaluated levels of inhibin B, which is Sertoli cell and germ cell biomarker. Results of the studies are summarized in **Table 7**.

**Table 7:** Summary of studies that evaluated postnatal EDC exposure and adult reproductive hormone levels.

EDC class	Reference	Matrix	Study design	Chemicals/ congeners reported	N of subjects	Country	Association between chemical levels and reproductive hormone levels
**Phenols: BPA**							
Adoamnei 2018	(164)	Urine	Cross-sectional	BPA	215 university students, aged 18-23 y	Spain	Positive association with serum LH No associations with other reproductive hormone levels
Galloway 2010	(244)	Urine	Cross-sectional	BPA	307 men from general population, aged > 20 y	Italy	Positive association with serum TT No association with E2, SHBG and FT
Hanaoka 2002	(245)	Urine	Cross-sectional	BPA	42 occupationally exposed and 42 non-exposed men Mean age, 37 y	Japan	FSH level was lower in the exposed group than that in the control. No differences in LH and FT between the groups
Scinicariello 2016	(246)	Urine	Cross-sectional	BPA	134 male children, aged 6-11 y and 161 male adolescents, aged 12-19 y	USA	Negative association with TT
Lassen 2014	(172)	Urine	Cross-sectional	BPA	308 young men from general population (median age: 19 y)	Denmark	Men with BPA level above the lowest quartile had higher TT, LH, E2 and FT vs men in the lowest quartile.
Li 2014	(247)	Urine	Cross-sectional	BPA	1116 middle-aged and elderly men Median age 61.4±9.6	China	NS (in multivariate analysis)
Liang 2017	(248)	Urine	Cross-sectional	BPA	560 men, aged 18-55 y, who had at least one child	China	Among current smokers, men with detectable BPA levels had higher FSH and LH levels as compared with men with undetectable BPA levels.
Liu 2015	(249)	Urine	Cross-sectional	BPA Serum FSH, prolactin, E2, T, inhibin B, androstenedione, FT, SHBG and FAI	592 male workers, aged 16-63 y (mean age, 31.7 y)	China	Positive association between BPA and prolactin, E2 and SHBG levels Negative association between BPA and androstenedione level and FAI Men with a higher quartile of BPA had a lower inhibin B.
Manfo 2019	(250)	Urine	Cross-sectional	BPA	44 male farmers and 37 men living in the urban area, aged 18-59 y	Cameroon	Negative association between BPA level and FT and bioavailable testosterone levels Positive association between BPA level and E2/T ratio
Meeker 2010	(251)	Urine	Cross-sectional	BPA	167 men from an infertility clinic (mean age, 37 y)	USA	Negative association between BPA level and E2:T ratio Positive association between BPA level and FSH level and FSH:inhibin B ratio
Mendiola 2010	(169)	Urine	Cross-sectional	BPA	375 partners of pregnant women (mean age, 31.9 y)	USA	Negative associations between BPA and FAI levels, FAI/LH ratio Positive association between BPA and SHBG levels
Vitku 2016	(179)	Plasma and seminal fluid	Cross-sectional	6 BPA congeners	191 men attending fertility clinic mean age 35.8 y	Czech	*Plasma BPA* Negative association with DHT, T/E2 ratio NS (TT level) *Seminal BPA* NS (TT, DHT levels, T/E2 ratio)
Zhou 2013	(252)	Serum	Cross-sectional	BPA	290 male workers (most were < 40 y)	China	Positive association between BPA and SHBG levels. Negative association between BPA and androstenedione, FT and FAI.
Zhuang 2015	(253)	Serum	Cross-sectional	Serum BPA Serum SHBG, TT, inhibin B, androstenedione	281 male workers exposed to BPA (mean age 34.1 y) 278 males not exposed to BPA (mean age 32.8 y)	China	Men exposed vs not exposed to BPA: no difference in SHBG, TT, inhibin B and androstenedione Men exposed to BPA of > 5y compared to exposure <5y: higher serum BPA and SHBG but lower serum androstenedione. BPA level of > 18.75 ng/mL was associated with lower androstenedione level and higher SHBG level compared with groups having lower BPA level.
**Flame retardants**							
Albert 2018	(184)	Hair	Cross-sectional	8 PBDE congeners	153 healthy men, aged 18-41 y)	Canada	NS
Guo 2018	(254)	Serum	Cross-sectional	sum of 13 PBDE congeners Sum of 8 new flame retardants	26 exposed men (residents from an e-waste dismantling region) and 25 non-exposed men Age 46−65 y	China	Sums of flame retardants showed positive association with T levels and negative association with FSH levels (the latter finding only with the sum of new flame retardants). No significant association with LH levels.
Makey 2016	(255)	Serum	Cross-sectional and longitudinal	11 PBDE congeners	27 healthy adult men Mean age = 41 y	USA	Negative association with inhibin B, positive association with FSH (in men aged 40 years or above). NS (with TT, Free T, prolactin, LH, FAI and SHBG)
Toft 2014	(236)	Serum	Cross-sectional	BDE-28, 47, 99,100, 153, 154 and 183, and BB-153	299 partners of pregnant women Median age: Greenland, 32.1 y Poland, 29.6 y Ukraine, 26.1 y	Greenland, Poland and Ukraine	BDE-47 and BDE 153: NS (no consistent associations across countries)
Turyk 2008	(256)	Serum	Cross-sectional	8 PBDE congeners	308 adult men (fish consumers) Mean age = 59 y	USA	BDE-47 was positively associated with testosterone levels in the tertile analysis. NS (with SHBG or SHBG-bound testosterone)
**PCBs**							
Vitku 2016	(179)	Plasma	Cross-sectional	6 PCB congeners	191 men attending fertility clinic mean age (SD) = 35.8 ± 5.6 y	Czech	Sum of PCB congeners: negative association with plasma TT, FT, FAI, DHT levels
Giwercman 2006	(257)	Serum	Cross-sectional	CB-153	Swedish fishermen (n=184, mean age 47 y) Greenland (n = 258, mean age 31 y) Poland (n = 113, mean age 31 y) Kharkiv, Ukraine (n = 194, mean age 27 y)	Sweden Greenland Poland Ukraine	Swedish fishermen: NS Greenland: positive association between CB-153 and LH levels Poland: lower FT in the third highest CB-153 group as compared with the lowest group Ukraine: positive association between CB-153 and SHBG and LH levels Pooled data set from all 4 centers: NS
Guo 2018	(254)	Serum	Cross-sectional	sum of 7 PCB congeners	26 exposed men (residents from an e-waste dismantling region) and 25 non-exposed men (age 46-65 y)	China	sum of PCBs: NS (with LH, FSH or T)
Petersen 2015	(227)	Serum	Cross-sectional	PCB28,105,118,156,52,101,153,138,180	266 fertile men Median age, 34.8 y	Faroe Island	Positive association between PCB and T/E2 ratio, SHBG and FSH levels
Petersen 2018	(220)	Serum	Cross-sectional	9 PCB congeners	263 Faroese men (24-26 y)	Faroe island	Positive association between ∑PCBs and SHBG, LH, TT and T/E2 ratio
Richthoff 2003	(228)	Serum	Cross-sectional	CB-153	305 men from general population, aged 18-21 y	Sweden	Negative associations between CB-153 levels and testosterone:SHBG ratio
**Phthalates**							
Albert 2018	(184)	Urine	Cross-sectional	Phthalate metabolites	153 healthy men, aged 18-41 y	Canada	NS
Al-Saleh 2019	(258)	Urine	Cross-sectional	8 phthalate metabolites	599 men attending IVF clinic Median age, 36.2 y	Saudi Arabia	Negative association between MiBP and TT, between %MEHP and T/LH and T/E2 and between MEHHP and FSH Positive association between MEP and E2 and between %MEHP and FSH and LH
Axelsson 2015	(185)	Urine	Cross-sectional	10 phthalate metabolites	314 men from general population, aged 17-20 y	Sweden	In adjusted models, Negative associations between %MEHP and T and FT No association between other metabolites and TT, FT, LH, FSH, E2 or SHBG
Chang 2015	(259)	Urine	Case-control study	Urinary concentrations of 11 phthalate metabolites	176 Infertile men from infertility clinic and fertile men (mean age, 34.2 y)	Taiwan	Urinary MnBP, MEHP and mono-2-ethyl-5-carboxy pentyl phthalate: infertile > fertile men Negative association between urinary MMP, MiBP, MEHP, MEHP% and serum TT Negative association between urinary MiBP, MBzP, MEHP, MEHP% and serum FT Negative association between urinary MMP, MEHP, MEHP% and TT:LH ratio Negative association between urinary MMP, MiBP, MnBP, MBzP, MEHP and FAI
Joensen 2012	(188)	Urine	Cross-sectional	14 urinary phthalate metabolites	881 men from general population (median age, 19.1 y)	Denmark	FAI: 15% lower for men in the highest %MiNP quartile vs lowest quartile FAI: 9% lower in the highest %MEHP quartile T/LH, T/FSH: negative association with %MEHP, %MiNP %MEHP had negative association with TT, FT, T/E2
Henrotin 2020	(260)	Urine	Short longitudinal	Urinary OXO-MINP, CX-MINP, OH-MINP	97 male workers (mean age, 44.5 y)	France	Urinary OXO-MINP had negative association with TT
Chen 2017	(261)	Urine	Cross-sectional	7 urine phthalate metabolites	786 subjects, aged 12-30 y, from general population	Taiwan	Negative association between urinary MEHP and T in men aged 20-30 y
Duty 2005	(262)	Urine	Cross-sectional	phthalate metabolites	295 men aged 18 to 54 y from andrology laboratory	USA	Negative association between MBzP and FSH levels
Jurewicz 2013	(189)	Urine	Cross-sectional	Urinary phthalate metabolites	269 men attending infertility clinic and had normal sperm concentration or slight oligozoospermia (mean age, 32 y)	Poland	Negative association between urinary MEHP level and TT level
Han 2014	(190)	Urine	Cross-sectional	Urinary levels of MBP MEP MEHP MBzP PA Total PA	232 men from 1 reproductive center (mean age, 33 y)	China	NS (TT, E2, LH, FSH, FAI)
Lenters 2015	(263)	Serum	Cross-sectional	6 phthalate metabolites	602 male partners of pregnant women Mean age: Greenland, 31.3 y Poland, 30.3 y Ukraine, 27.9 y	Greenland Poland Ukraine	Negative association between DiNP metabolites and TT
Meeker 2009	(264)	Urine	Cross-sectional	MEP MBP MBzP MEHP MEHHP MEOHP DEHP	Men of infertile couples Age 18-55 y	USA	Negative associations between MEHP level and T and E2 levels Positive associations between MEHP level and FAI and T:E2 ratio
Meeker 2014	(265)	Urine	Cross-sectional	13 phthalate metabolites	707 men aged 20-80 y	USA	Negative association between urinary DEHP metabolites, MBP and T among men aged 40–60
Mendiola 2011	(266)	Urine	Cross-sectional	11 phthalate metabolites	425 male partners of pregnant women (mean age 32.2 y)	USA	Negative associations between phthalate metabolites (MEHP, MEHHP, MEOHP, ∑DEHP) and FAI Negative association between MEHP and FAI/LH Positive association between MEHP and SHBG
Pan 2015	(195)	Urine	Cross-sectional	14 phthalate metabolites	1066 male partners of infertile couples (median age 29 y)	China	Negative associations of MBP and MiBP with TT, FAI, FT and LH levels Negative associations of MEHP and %MEHP with INSL3 level
Pant 2014	(215)	Seminal fluid	Cross-sectional	Seminal fluid for phthalate	85 fertile men and 193 men from infertile couples, aged 21-40 y	India	Negative association between DBP, DEHP and T level
Pant 2014	(193)	Seminal fluid	Cross-sectional	DEHP DBP DEP	60 male partners of couples attending the andrology laboratory Age 21-40 y	India	Negative associations between DEHP and T level and between DBP and T level
Specht 2014	(199)	Serum	Cross-sectional	5OH-MEHP oxo-MEHP 5cx-MEPP 7OH-MMeOP 7oxo-MMeOP 7cx-MMeOP	589 male partners of pregnant women Mean age: Greenland, 31 y Poland, 30.3 y Ukraine, 26.5 y	Greenland Poland Ukraine	Negative association between TT and - Proxy-MEHP 5OH-MEHP 5CX-MEPP Proxy-MiNP 7OH-MMeoP 7cx-MMeHP Negative association between SHBG and Proxy-MiNP and 7cx-MMeHP Negative association between T/LH ratio and 5OH-MEHP
Wang 2016	(198)	Seminal fluid	Cross-sectional	8 phthalate metabolites	Male partners of subfertile couples Semen samples (n = 687) Blood samples (n = 342)	China	NS
Wang 2016	(267)	Urine	Cross-sectional	8 phthalate metabolites	483 male partners of couples attending fertility clinic Who had serum reproductive hormone measurement Mean age, 32.1 y	China	Negative association between MEHP, DEHP and E2, TT and FT levels
Woodward 2020	(268)	Urine	Cross-sectional	19 phthalate metabolites	1420 men from general population, aged ≥20 y Median age, 47 y	USA	Age 20-39 y Positive association between ∑DEHTP and TT Negative association between ∑LMW phthalates and FT Age 40-59 y Positive association between ∑LMW phthalates and FT Negative association between ∑DINCH and TT Age ≥60 y Negative association between ∑DEHP, ∑DINCH and TT, between ∑DEHP, ∑DINP and E2 and between ∑HMW, ∑DEHP and FT
**Perfluorinated compounds (PFCs)**							
Den Hond 2015	(269)	Serum	Cross-sectional	PFOA PFOS	Men from fertility clinics 40 cases with total motility count (TMC) < 20 million 80 controls (TMC ≥ 20 million) Mean age: cases, 31.6 y controls, 34.1 y	Belgium	NS (FSH, LH, SHBG, total 17β-estradiol, inhibin B and total testosterone)
Lewis 2015	(219)	Serum	Cross-sectional	PFASs Serum T	857 males from general population Age 12-80 y	USA	NS with T
Petersen 2018	(220)	Serum	Cross-sectional	Blood for PFASs	263 Faroese men (24-26 y)	Faroe island	Positive association between PFOS and SHBG and LH
Joensen 2009	(218)	Serum	Cross-sectional	PFHxS, PFHpA, PFOA, PFOS, PFOSA, PFNA, PFDA, PFUnA, PFDoA, PFTrA	105 men (53 men with the highest T level and 52 men with the lowest T level) Median age, 19 y	Denmark	NS (T, E2, SHBG, FSH, LH, inhibin B, FAI, T/LH, FAI/LH, E2/T and inhibin/FSH)
Joensen 2013	(221)	Serum	Cross-sectional	14 PFASs	247 men from general population Mean age 19.6 y	Denmark	Negative association between PFOS and TT, FT, FAI, FT/LH, FAI/LH, T/LH ratios Negative association between PFNA and E2
Raymer 2012	(222)	Plasma, seminal fluid	Cross-sectional	PFOS, PFOA	256 men came for fertility assessment Median age, 41 y	USA	Positive association between plasma PFOA and LH levels No association between seminal PFOA, PFOS and any hormones (E2, Prolactin, FSH, FT, TT, TSH, LH, T3, T4)
Specht 2012	(270)	Serum	Cross-sectional	4 PFASs	604 men Median age: Greenland: 30.6 y Poland: 29.6 y Ukraine: 25.1 y	Greenland, Poland and Ukraine	No association with TT, E2, FSH, LH, inhibin B and SHBG
**Triclosan and parabens**							
Scinicariello 2016	(246)	Urine	Cross-sectional	Triclosan parabens	134 male children, aged 6-11 y and 161 male adolescents, aged 12-19 y	USA	No association with TT
Den Hond 2015	(269)	Urine	Cross-sectional	Triclosan	163 men from fertility clinic, aged < 50 y	Belgium	Positive association between triclosan and LH Negative association between triclosan and inhibin B
Jurewicz 2017	(56)	Urine	Cross-sectional	Parabens	315 men from infertility clinic Median age, 31.6 y	Poland	Negative association between parabens and TT
Meeker 2011	(182)	Urine	Cross-sectional	Parabens	167 male partners attending infertility clinic who had hormone results Mean age, 36.7 y	USA	NS
**Pesticides**							
Aguilar-Gardu ño 2013	(271)	Urine	Longitudinal	6 DAP metabolites	136 male floriculture workers (age 18-52 y)	Mexico	Positive association between urinary DAP levels and serum FSH and prolactin levels Negative association between urinary DAP levels and serum TT and inhibin B levels Negative association between DETP and LH levels
Bornman 2018	(272)	DDT and DDE uptake	Cross-sectional	DDT, DDE uptake	535 men, aged 18-40 years Exposed and non-exposed to indoor residual spraying	South Africa	Men with DDE uptake had higher TT, FT, bioavailable T and estradiol and lower FSH vs men with no DDE uptake. Men with DDT uptake had higher FT and bioavailable T, estradiol and lower FSH and LH vs men with no DDT uptake. Men with DDT or DDE levels in the highest quartile had higher TT vs men in other categories. Men with DDE in the highest category had higher E2 and lower FSH vs men in other categories.
Den Hond 2015	(269)	Serum	Cross-sectional	HCB	163 men from fertility clinics, aged < 50 y	Belgium	Positive association between HCB and SHBG levels Negative association between HCB and FT and free E2
Giwercman 2006	(257)	Serum	Cross-sectional	p,p’-DDE	Swedish fishermen (n=184) Greenland (n = 258) Poland (n = 113) Kharkiv, Ukraine (n = 194)	Sweden Greenland Poland Ukraine	Swedish fishermen: NS Greenland: Positive association between p,p’-DDE and FT The highest p,p’-DDE group had higher inhibin B. Poland: NS Ukraine: positive association between p,p’-DDE and SHBG and LH p,p’-DDE: negative association with inhibin B Pooled dataset from all 4 centers: positive association between p,p’-DDE and FSH
Han 2008	(273)	Urine	Cross-sectional	3-PBA	212 men Mean age 29.4 y	China	Positive association between 3-PBA and LH levels Negative association between 3-PBA and E2 levels
Martin 2002	(274)	Plasma	Cross-sectional	DDE	137 men Mean age 60 y	USA	NS (TT, bioavailable T, FAI, DHT)
Miranda-Cantreras 2013	(200)	Erythrocyte acetylcholinesterase (AChE) and plasma butyrylcholinesterase	Cross-sectional	Erythrocyte acetylcholinesterase (AChE) and plasma butyrylcholinesterase	35 healthy farm male workers (unexpected group) and 64 male agricultural workers (exposed group)	Venezuela	NS
Ghafouri-Khosrowshahi 2019	(241)	Serum	Cross-sectional	Serum butyrylcholinesterase (BChE) activity	30 rural farmers and 30 urban men, aged 20-40 years	Iran	Rural farmers had lower BChE activity. Rural farmers had lower LH and higher TT than those of the urban men. FSH levels: no difference
Panuwet 2018	(275)	Urine	Cross-sectional	Urinary levels of organophosphates, pyrethroids, selected herbicides, and fungicides	133 farmers (mean age 40 y)	Thailand	Negative association between 2,4-D and TT Positive association between DEP, DEDTP and total testosterone
Meeker 2006	(276)	Urine	Cross-sectional	TCPY (metabolite of chlorpyrifos) and 1N (metabolite of carbaryl and naphthalene)	268 male partners of couples visiting infertility clinic	USA	Negative associationbetween TCPY, 1N and T level
Meeker 2008	(277)	Urine	Cross-sectional	TCPY, 1N and 2N	322 male partners of couples attending infertility clininc	USA	Negative association between TCPY and E2 levels
Meeker 2009	(278)	Urine	Cross-sectional	3PBA and cis-DCCA and trans-DCCA	161 men from an infertility clinic (age 18-54 y)	USA	Positive association between 3PBA, cis-DCCA, trans-DCCA levels and FSH and LH levels Negative association between cis-DCCA, trans-DCCA levels and inhibin B levels
Melgarejo 2015	(201)	Urine	Cross-sectional	6 urinary DAP metabolites	116 men, 25-38 years old (median age 35.1 y)	Spain	Negative association between DEDTP levels and serum TT/LH levels Positive association between DEDTP levels and serum LH and FSH levels
Omoike 2015	(279)	Urine	Cross-sectional	Organophosphate metabolites (TCPY and 6 DAPs)	356 men, aged 20-55 y Median age, 37 y	USA	Negative association between DEP and T levels Positive association between TCPY and E2 levels
Radwan 2014	(206)	Urine	Cross-sectional	Pyrethroid metabolites: 3-PBA CDCCA TDCCA DBCA	334 men from infertility clinic Mean age, 32.2 y	Poland	Negative association between levels of TDCCA (>50^th^) and T
Specht 2015	(216)	Serum	Cross-sectional	HCB	589 fertile men Median age: -Greenland 30.6 y - Poland 29.6 y - Ukraine 25 y	Greenland Poland Ukraine	Positive association between HCB and SHBG Negative association between HCB and FAI
Yoshinaga 2014	(280)	Urine	Cross-sectional	3-PBA	322 male university students, aged 18-24 y	Japan	NS

NS, no statistically significant association.

Only statistically significant findings are shown.

##### 2.5.2.1 Phenols: Bisphenol A

Associations between BPA levels and reproductive hormone levels were examined in 14 cross-sectional studies (**Table 7**), and they showed variable results. Eleven studies analyzed BPA level in urine samples, two studies analyzed BPA level in serum (252, 253), and one study measured BPA level in plasma and seminal plasma (179). An association between BPA level and serum testosterone level was not significant in most studies (164, 169, 179, 247–253). Two studies have demonstrated a positive association between BPA level and serum total testosterone level (172, 244) and only one study showed a negative association (246). Some studies did not show significant association between BPA and LH levels (169, 245, 250, 251), whereas some showed a significant positive association (164, 172, 248). Studies on the relationship between BPA and FSH levels have also shown mixed results – most studies did not show any significant correlation (164, 169, 172, 247, 250, 252), while two studies showed a positive association (248, 251). Some studies also evaluated inhibin B level, which showed no significant association with BPA level (164, 169, 172, 252, 253).

##### 2.5.2.2 Flame Retardants

Several studies have evaluated associations between flame retardant levels in serum or hair and reproductive hormone levels in adult men. Two studies suggested a positive association with testosterone levels (254, 256). One small study suggested a negative association with inhibin B levels (255). In contrast, two large studies found no consistent or significant association between reproductive hormone levels and flame retardant levels (184, 236).

##### 2.5.2.3 Phthalates

Experimental studies showed that phthalates had a variety of effects on the HPG axis function in male rats, including low FSH and LH levels as well as high or low GnRH and testosterone levels [reviewed in Hlisníková 2020 (281)]. Phthalates can also disturb testicular hormone production by altering steroidogenic enzyme activity, including decreased or increased levels of CYP11a1, Hsd3b, Hsd17b enzymes and decreased levels of CYP17a1 enzyme, changes in steroidogenic acute regulatory protein (StAR) amount (281).

Epidemiological studies, most of which were cross-sectional, have shown inconsistent results on the association between phthalate and reproductive hormone levels. Phthalates or phthalate metabolites were measured in urine in most studies (184, 260–262, 264, 265, 267, 282), in serum in three studies (199, 263) and in seminal fluid in three studies (193, 198, 215). Numerous studies showed an association between levels of phthalates or phthalate metabolites and low serum total or free testosterone levels (189, 195, 258, 260, 261, 264, 265, 267, 268, 282, 283), and one study also found a concurrent low LH level (195), suggesting an impaired LH secretion as a cause of low testosterone level. Some studies assessed levels of serum inhibin B, which reflects Sertoli and germ cell function and/or number, and they showed that there was no association between phthalate and inhibin B levels (188, 199, 263, 266, 284, 285), except for a negative association between urinary MiBP levels and serum inhibin B levels which was found in a Chinese study (259).

##### 2.5.2.4 Polychlorinated Biphenyls

Some studies have demonstrated a negative association between PCB exposure and serum total testosterone levels (179, 286). Some studies have shown an association with low free testosterone level (179, 228, 257, 287), which might be due to an associated increased SHBG level in some studies (228, 257). Most studies did not show any significant associations with FSH and LH, except for two studies. Lin et al. found a negative association between CB52, CB209 and LH level and a positive association between CB44 and LH level (288), while CB170 level was positively associated with total testosterone levels (288). Petersen et al. reported a positive association between PCB level and serum FSH level (227). Giwercman et al. found no association between PCB level and serum FSH and inhibin B levels in Sweden, Greenland, Poland, and Ukraine, suggesting no disturbance in the hypothalamic-pituitary-Sertoli cell axis (257). A study from China found either no significant association between serum PCB levels and reproductive hormone levels (254). Overall, evidence suggests a link between PCB exposure and disturbed hypothalamic-pituitary-gonadal axis in men, particularly low serum testosterone level.

##### 2.5.2.5 Perfluorinated Compounds

Cross-sectional studies on the link between perfluorinated compound and reproductive hormone levels in adult men have shown inconsistent results. Four studies did not show any significant association (218, 219, 269, 270). Only one study by Joensen et al. showed negative associations with total and free testosterone levels, free androgen index, free testosterone/LH, total testosterone/LH and free androgen index/LH (289). Positive association between serum PFOS and LH was shown in one study (220) and between plasma PFOA and LH in another study (222).

##### 2.5.2.6 Pesticides

Several studies have examined the association between different pesticide exposure and reproductive hormone levels in adult (200, 201, 206, 216, 241, 257, 269, 271–280). The studies have shown mixed results, which are summarized in **Table 7**.

### 2.6 Testicular Cancer

Testicular germ cell tumors (TGCTs) are relatively rare - accounting for about 1% of cancers in men. However, they are the most common cancer in young adult men (290, 291). Their prevalence has been increasing in many Western countries (292). The main cause of this adverse trend is still unclear, but it has been proposed that EDCs might have a role (11). Testicular cancer appears to have a fetal origin, although it usually manifests after puberty when gonadotropin stimulation has started (293). Testicular cancer, cryptorchidism and hypospadias have similar prenatal risk factors and men with a history of cryptorchidism or hypospadias have an increased risk of testicular cancer (11).

Most of the studies that investigated the relationship between EDC exposure and testicular cancer used data on self-reported exposures or the occupational history or a history of chemical use without showing the chemical levels. Studies which reported EDC concentrations are scarce. Many studies are case-control studies. In addition, cohort studies evaluating the association between prenatal exposure levels and testicular cancer occurrence are lacking. Therefore, the cause-and-effect relationship is inconclusive.

#### 2.6.1 Early Life EDC Exposure

Even though TGCTs are most commonly diagnosed between the ages of 15-40 years, there is evidence supporting the hypothesis that prenatal exposure to EDCs has a role in the development of testicular cancer.

A Swedish study of 44 TGCT case mothers and 45 control mothers found that the concentrations of the sum of PCBs, sum of PBDEs, hexachlorobenzene (HCB), cis- and transnonachlordane and sum of chlordanes were higher in case mothers than in control mothers (294, 295), suggesting a link between prenatal exposure to these chemicals and the development of TGCTs. The chemical levels of maternal blood samples were analyzed when the sons were diagnosed with testicular cancer. Chemical measurements were not performed in the blood taken during pregnancy; therefore, the timing of chemical exposure was unclear. However, these findings suggest a link between testicular cancer and chemical exposures, since these organochlorines have very long half-life and can stay in human body for several years.

#### 2.6.2 Concurrent EDC exposure

##### 2.6.2.1 Pesticides

A nested case-control study of 49 TGCT cases and 51 controls in Norway used pre-diagnostic serum samples, and no significant difference in the levels of oxychlordane, trans-nonachlor, and total chlordanes between the cases and controls was reported (296).

To date, five case-control studies have examined relationship between pre-diagnostic serum levels of p,p’-DDE and TGCTs. Two studies found higher levels of p, p’-DDE in TGCT group than those in the controls. A study among US servicemen (297) and a hospital-based study in Italy showed that the TGCT cases had significantly higher p,p’-DDE levels than those of the controls (298). A Swedish study and a Norwegian study found a tendency to higher serum p,p’-DDE levels among the TGCT cases as compared with controls; however, the difference was not statistically significant (295, 296). Another US study did not show an association between TGCT and serum DDE (299).

##### 2.6.2.2 Polychlorinated Biphenyls

Three studies have investigated the associations between PCB exposure and the occurrence of TGCTs. A study in Norway found that the levels of some PCB congeners (PCB-99, -138, -153, -167, -183 and -195) were significantly higher in seminoma cases and the levels of some PCB congeners (PCB-44, -49, -52) were significantly lower in seminoma cases than in the controls (296). A case-control study in Sweden found no difference between the levels of PCBs between TGCT cases and controls (300). An Italian study found that men with detectable levels of total polychlorinated organic compounds (PCB congeners (PCB-31, -28, -52, -77, -153, -126, -180, -169, -170) and hexachlorobenzene) had increased risk of TGCTs as compared with men with undetectable levels (301). In contrast, a US study found that PCB-118, PCB-138, PCB-153, PCB-156, PCB-163, PCB-170, PCB-180, PCB-187 levels were associated with a decreased risk of TGCT and PCB-99, PCB-101, PCB-183 were not associated with the occurrence of TGCT (302).

In summary, studies on the role of prenatal EDC exposure on TGCTs are scarce. Studies evaluating the role of concurrent EDC exposure on TGCTs have shown mixed results. However, significant associations between EDC exposure and testicular cancer have been shown at least in some studies. More studies are needed to further assess these connections.

## 3 Discussion

There has been a growing research interest in the potential health risk of EDCs during recent years. Experimental studies support the role of EDC exposure in the occurrence of male reproductive health problems. Results from epidemiological studies are mixed, however, evidence suggests a link between some EDC exposures and adverse male reproductive health. Maternal exposure to some EDCs during pregnancy has, at least in part of the studies, been associated with congenital urogenital anomalies, i.e., cryptorchidism and hypospadias, and low semen quality, altered HPG hormone levels and testicular cancer in adult men. The evidence for the link to the adverse adult male reproductive health is derived from a small number of studies. The association of concurrent exposure to some EDCs in adulthood with low semen quality, low serum testosterone levels and testicular cancer has been reported, although the results are not consistent.

Human studies on the association between exposure to environmental EDCs and male reproductive health are challenging because of a number of factors. First, we are continuously exposed to a mixture of different chemicals, which is different from many experimental studies that evaluated the effect of one chemical at a time. In addition, the level of exposure in animal models can be higher than human exposure in real life. Results from experimental studies are not always repeatable in human studies. Second, the exposure starts already at the embryonic period or even before that, since paternal exposure to environmental and lifestyle factors may change sperm epigenome and recent studies suggest that such changes may be the link between paternal exposures and offspring health (303, 304). Furthermore, the critical period for exposure may vary for different reproductive outcomes, since for instance hypospadias is caused by a defect in fetal development of penile urethra, but sperm production capacity is determined by the number of Sertoli cells and these cells divide fast during fetal development but also postnatally and at the beginning of puberty (133, 305). Therefore, the timing of exposure measurement may affect the results on the association between EDC exposure and male reproductive health. Third, participant settings – men from general population, men who had occupational exposure to EDCs, or men who lived in the areas of accidental chemical leakage - also influence the results. Studies on the effects of accidental chemical leakage have usually shown a negative impact on semen quality or male reproductive hormone levels, while studies in men from general population are more likely to show mixed results. Men recruited from an infertility clinic, men from general population and men at a different age possibly show dissimilar association to chemical exposures. In addition, differences in exposure levels between study population may influence the observed associations. Fourth, a cross-sectional study examines the relationship between chemical exposure and semen quality or reproductive hormones at one point of time. For a chemical with a short half-life, chemical measurement at a single point might not reflect the real level of exposure in long-term. In addition, a significant correlation observed in cross-sectional study does not indicate a cause-and-effect relationship. Lastly, studies on the association between prenatal EDC exposures and adult male reproductive outcomes, including semen quality, serum reproductive hormone levels and testicular cancer need long period of follow-up, and are therefore difficult to conduct. In addition, prenatal EDC exposure is also followed by postnatal exposure from birth to adulthood.

More studies on the effects of maternal EDC exposures on the sons’ semen quality and reproductive hormone levels, and more results from birth cohort studies would be beneficial. Role of paternal EDC exposure during pre-conception, particularly epigenetic studies, is a topic that needs to be studied further.

## Author Contributions

All authors listed have made a substantial, direct, and intellectual contribution to the work and approved it for publication.

## Funding

This work was supported by the Academy of Finland (308065), Sigrid Juselius Foundation, Novo Nordisk Foundation, Special governmental funds for Turku University Hospital, Finnish Cultural Foundation, Jalmari and Rauha Ahokas Foundation, Kirsten and Freddy Johansen’s Fund, Juho Vainio Foundation, Foundation for Pediatric Research and Danish Innovation Funds.

## Conflict of Interest

The authors declare that the research was conducted in the absence of any commercial or financial relationships that could be construed as a potential conflict of interest.

## Publisher’s Note

All claims expressed in this article are solely those of the authors and do not necessarily represent those of their affiliated organizations, or those of the publisher, the editors and the reviewers. Any product that may be evaluated in this article, or claim that may be made by its manufacturer, is not guaranteed or endorsed by the publisher.

## Glossary

**Table:**

AGD	anogenital distance
AhR	aryl hydrocarbon receptor
β-HCH	beta-hexachlorocyclohexane
beta-HCCH	beta-hexachlorocyclohexane
BBP	butyl benzyl phthalate
BP	butyl paraben
BPA	bisphenol A
BzP	benzyl paraben
CASA	computer-assisted semen analysis
CDCCA	cis-3-(2,2-dichlorovinyl)-2,2-dimethylcyclopropane carboxylic acid
2,4-D	2,4-dichlorophenoxyacetic acid
2,4-DDD	2,4-dichlorodiphenyldichloroethane
4,4′-DDD	1,1-bis(4-chlorophenyl)-2,2-dichloroethane, 4,4′-dichlorodiphenyldichloroethane
DAPs	dialkylphosphates
DBCA	cis-2,2-dibromovinyl-2,2-dimethylcyclopropane-1-carboxylic acid
DBP	dibutylphthalate
DBT	dibutyltin
DCCA	3-(2,2-dichlorovinyl)-2,2-dimethylcyclopropane carboxylic acid
DDD	4,4′-dichlorodiphenyldichloroethane
DDE	dichlorodiphenyldichloroethylene
4,4′-DDE	2,2-bis(4-chlorophenyl)-1,1-dichloroethene
DDT	dichlorodiphenyltrichloroethane
4,4′-DDT	1,1,1-trichloro-2,2-bis(p-chlorophenyl)ethane
p,p’-DDT	1,1,1-trichloro-2,2-bis(4-chlorophenyl)ethane
DEDTP	diethyldithiophosphate
DEHP	di(2-ethylhexyl) phthalate
DEP	diethyl phthalate
DETP	diethylthiophosphate
3,4 DHB	3,4-dihydroxy benzoic acid
DiNP	diisononyl phthalate
DL-PCBs	dioxin-like polychlorinated biphenyls
DMDTP	dimethyldithiophosphate
DMP	dimethylphosphate
DMTP	dimethylthiophosphate
E2	estradiol
EP	ethyl paraben
ER	estrogen receptor
FAI	free androgen index
FSH	follicle-stimulating hormone
FT	free testosterone
GW	gestational week
4-HB	4-hydroxy benzoic acid
HCB	hexachlorobenzene
HCE	heptachloroepoxide
HCH	hexachlorocyclohexane
HP	heptyl paraben
HPG	hypothalamic-pituitary-gonadal
hsd3b	3 beta-hydroxysteroid dehydrogenase
hsd17b	17β-Hydroxysteroid dehydrogenase
iBuP	isobutyl paraben
INSL3	Insulin-like peptide 3
LH	luteinizing hormone
MAA	methoxyacetic acid
mBP	MBP, monobutylphthalate
MBT	monobutyltin
MBzP	mono-benzyl phthalate
MCiOP	mono-carboxy-iso-octyl phthalate
MCPP	mono-3-carboxypropyl-phthalate
MECPP	mono(2-ethyl-5-carboxypentyl) phthalate
MEHP	monoethylhexyl phthalate
MEHHP	mono(2-ethyl-5-hydroxyhexyl) phthalate
MEOHP	mono(2-ethyl-5-oxohexyl) phthalate
MEP	monoethyl phthalate
MBzP	monobenzyl phthalate
MHiNP	mono-hydroxy-iso-nonyl phthalate
MiBP	monoisobutyl phthalate
MiNP	monoisononyl phthalate
MnBP	mono-n-butyl phthalate
MOiNP	mono-(oxo-iso-nonyl) phthalate
MP	methyl paraben
MPW	male programming window
1N	1-naphthol
2N	2-naphthol
OCDF	octachlorodibenzofuran
OH-EtP	ethylprotocatechuic acid
OH-MeP	methyl-protocatechuic acid
OTCs	organotin compounds
PA	phthalic acid
3-PBA	3-phenoxybenzoic acid
PBBs	polybrominated biphenyls
PCBs	polychlorinated biphenyls
PBDEs	polybrominated diphenyl ethers
PCDD/Fs	polychlorinated dibenzo-p-dioxins and dibenzofurans
PFDA	perfluorodecanoic acid
PFDoA	perfluorododecanoic acid
PFHpA	perfluoroheptanoic acid
PFHpS	potassium perfluoro-1-heptanesulfonate
PFHxS	perfluorohexane sulfonic acid
PFNA	perfluorononanoic acid
PFOA	Perfluorooctanoic acid
PFOS	perfluorooctanesulfonic acid
PFOSA	perfluorooctane sulfonamide
PFTrA	perfluorotridecanoic acid
PFUnA	perfluoroundecanoic acid
PhAA	phenoxyacetic acid
POPs	persistent organic pollutants
PP	propyl paraben
p,p’-DDE	p,p′-dichlorodiphenyldichloroethylene
SHBG	sex hormone-binding globulin
StAR	steroidogenic acute regulatory protein
T	testosterone
TBT	tributyltin
TCPY	3,5,6-trichloro-2-pyridinol
TDCCA	trans-2,2-(dichlorovinyl)-2,2-dimethylcyclopropane carboxylic acid
TEQ	toxic equivalent
TGCTs	testicular germ cell tumors
TPhT	triphenyltin
TSC	total sperm count
TT	total testosterone
UV	ultraviolet
Y	year
